# Anion-Dependent Strength Scale of Interactions in Ionic Liquids from X-ray Photoelectron Spectroscopy, Ab Initio Molecular Dynamics, and Density Functional Theory

**DOI:** 10.1021/acs.jpcb.4c00362

**Published:** 2024-05-10

**Authors:** Ekaterina Gousseva, Frances K. Towers Tompkins, Jake M. Seymour, Lewis G. Parker, Coby J. Clarke, Robert G. Palgrave, Roger A. Bennett, Ricardo Grau-Crespo, Kevin R. J. Lovelock

**Affiliations:** † Department of Chemistry, University of Reading, Reading RG6 6DX, U.K.; ‡ School of Chemistry, University of Nottingham, Nottingham NG7 2RD, U.K.; § Department of Chemistry, University College London, London WC1H 0AJ, U.K.

## Abstract

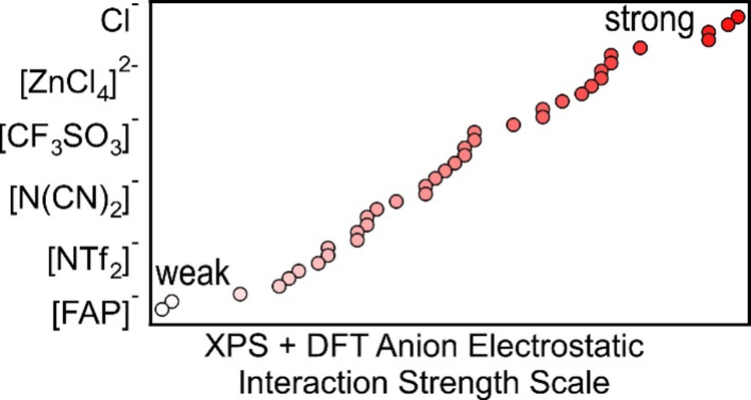

Using a combination of experiments and calculations, we have gained new insights into the nature of anion–cation interactions in ionic liquids (ILs). An X-ray photoelectron spectroscopy (XPS)-derived anion-dependent electrostatic interaction strength scale, determined using XPS core-level binding energies for IL cations, is presented here for 39 different anions, with at least 18 new anions included. Linear correlations of experimental XPS core-level binding energies for IL cations with (a) calculated core binding energies (ab initio molecular dynamics (AIMD) simulations were used to generate high-quality model IL structures followed by single-point density functional theory (DFT) to obtain calculated core binding energies), (b) experimental XPS core-level binding energies for IL anions, and (c) other anion-dependent interaction strength scales led to three main conclusions. First, the effect of different anions on the cation can be related to ground-state interactions. Second, the variations of anion-dependent interactions with the identity of the anion are best rationalized in terms of electrostatic interactions and not occupied valence state/unoccupied valence state interactions or polarizability-driven interactions. Therefore, the XPS-derived anion-dependent interaction strength scale can be explained using a simple electrostatic model based on electrostatic site potentials. Third, anion–probe interactions, irrespective of the identity of the probe, are primarily electrostatic, meaning that our electrostatic interaction strength scale captures some inherent, intrinsic property of anions independent of the probe used to measure the interaction strength scale.

## 1 Introduction

In ionic liquids (ILs), liquids composed solely of ions, anion–cation interactions are very important,^1,2^ as they heavily influence both macroscopic and mesoscopic properties including static (e.g., density, vapor pressure, surface tension, solubility) and transport properties (e.g., conductivity, viscosity, and chemical reactivity). The drivers for these properties need to be understood to push forward the many potential applications of ILs: electrochemical energy storage,^3−5^ metal electrodeposition,^6^ sensors,^7^ gas capture and storage,^8^ solvents for catalysis,^9^ and metal extraction and recycling.^10,11^ Therefore, understanding anion–cation interactions is vital. However, identifying and classifying anion–cation interactions is very challenging; experimental evidence is relatively scarce, and the size and complexity of IL anions and cations make quantum chemical studies of anion–cation interactions difficult.

**Figure 1:**
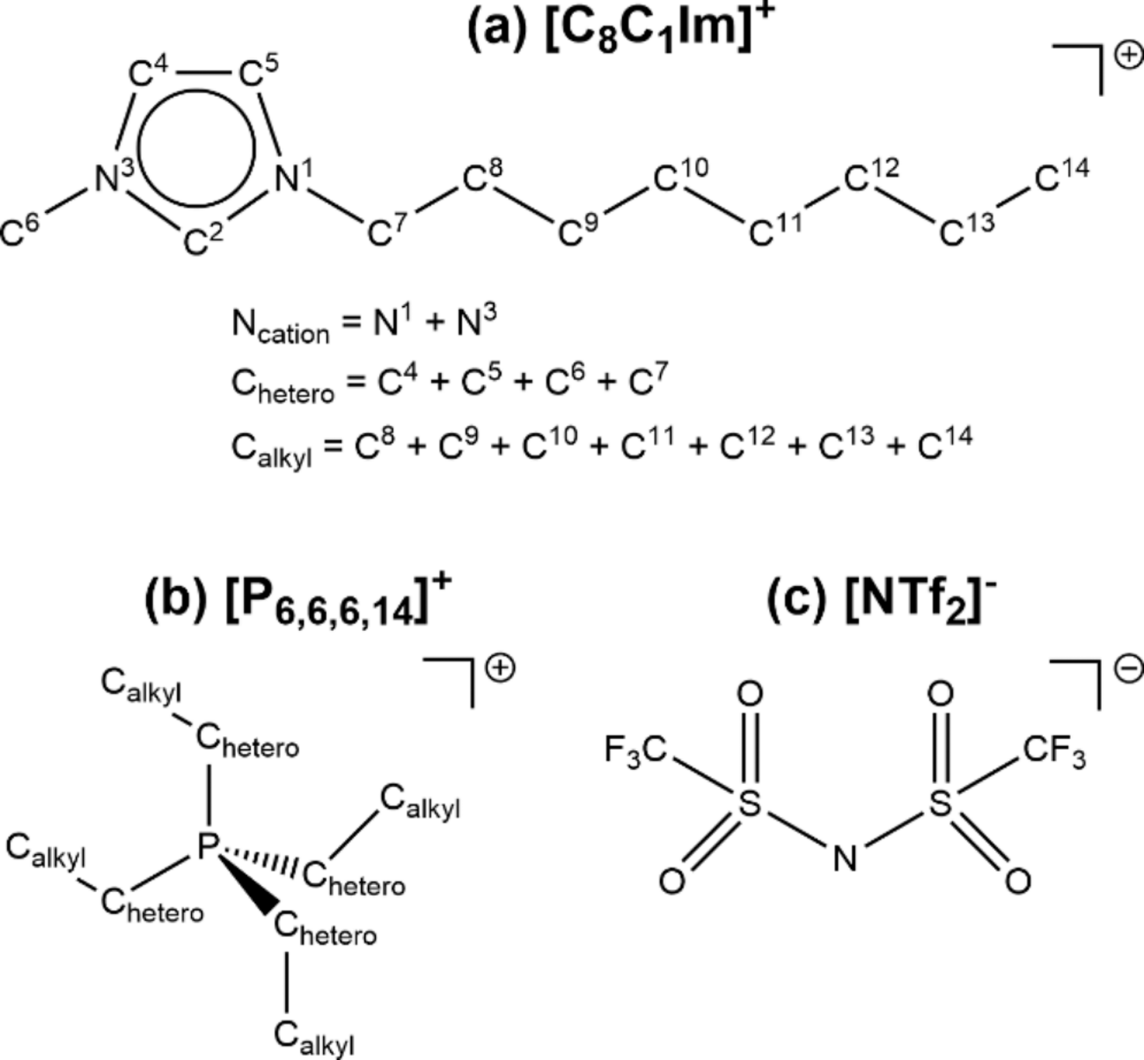
Structure of key ions with labels relevant to XPS: (a) 1-octyl-3-methylimidazolium = [C_8_C_1_Im]^+^, (b) tetradecyl(trihexyl)phosphonium = [P_6,6,6,14_]^+^, and (c) bis[(trifluoromethane)sulfonyl]imide = [NTf_2_]^−^.

Experimental X-ray photoelectron spectroscopy (XPS)^12−14^ of ILs with the cation 1-octyl-3-methylimidazolium ([C_8_C_1_Im]^+^, Figure 1a) and ∼25 different anions [A]^−^ has shown anion-dependent differences for experimental cation core binding energies, *E*_B_(cation core,exp.),^15−18^ giving an XPS-derived anion-dependent interaction scale. The same anion-dependent interaction scale has been demonstrated for a range of further organic cations, including both aromatic^15,19,20^ and nonaromatic (e.g., tetradecyl(trihexyl)phosphonium, [P_6,6,6,14_]^+^, Figure 1b).^21,22^ However, the nature of this anion-dependent interaction is not yet understood.

The anion-dependent *E*_B_(cation core,exp.) differences can be due to a ground-state effect (called the initial-state effect in the XPS community), which is related to the bonding/interactions, or due to the core-hole created by the photoemission process in XPS (called the final-state effect in the XPS community), which is related to relaxation of other core and valence electrons after the core-hole is created but before photoemission.^23,24^ In the initial-state interpretation, *E*_B_(cation core,exp.) differences can be understood in terms of the electrostatic site potentials and the charge potential model,^25,26^ where greater electron-withdrawing power of substituents/ligands/counterions surrounding an atom corresponds to larger *E*_B_ for that atom.^25^ In the final-state interpretation, *E*_B_(cation core,exp.) differences can be understood in terms of the ability of electrons surrounding the atom with the core-hole to react to the creation of that core-hole, akin to the polarizability of the neighboring atoms. Almost all experimental XPS studies of ILs have assumed that the initial-state interpretation holds.^15−22,27−64^ Calculated core binding energies, *E*_B_(core,calc.), for a small number of IL ion pairs in the initial-state approximation (without a core-hole)^31^ and the final-state approximation (with a core-hole)^37,65^ gave reasonable matches to experimental data, and comparisons of *E*_B_(core,exp.) and calculated atomic charges gave acceptable correlations.^15,38,43,44^ Recently, ab initio molecular dynamics (AIMD) calculations (to obtain structures that are more representative of ILs in the liquid phase) followed by single-point density functional theory (DFT) of [C_4_C_1_Im][SCN] suggested that initial-state effects dominated local variations of *E*_B_(core) within the IL. This conclusion was based on the fact that initial-state calculations gave excellent visual matches for the N 1s and final-state calculations for S 2p and N 1s in the anion [SCN]^−^ gave the same trends as initial-state calculations.^66^ However, no results were presented on anion-dependent interactions. Hence, whether the XPS-derived anion-dependent interaction strength scale is due to initial-state effects or final-state effects is still an open question.

The molecular origin of this XPS-derived anion-dependent interaction was suggested, based on gas phase ion pair calculations for three ILs ([C_8_C_1_Im]^+^ with [NTf_2_]^−^ (bis[(trifluoromethane)sulfonyl]imide, Figure 1c), [BF_4_]^−^, and Cl^–^), to be a ground-state anion-dependent anion-to-cation electron density donation through orbital mixing between the anion and cation, termed charge transfer.^15,67^ Experimental XPS results for the nonmethylated versus methylated imidazolium cations (at the C^2^ position, Figure 1a) ruled out a hydrogen bonding interaction to explain the XPS-derived anion-dependent interaction scale.^15^ However, an explanation based on larger-scale calculations of the molecular origin of the XPS-derived anion-dependent interaction scale is yet to be made.

Excellent linear correlations have been found between the XPS-derived anion-dependent interaction scale and a ultraviolet–visible (UV–vis) spectroscopy-derived anion-dependent hydrogen-bond acceptor interaction scale (Kamlet–Taft β values).^15,16^ A nonlinear relationship was reported for the XPS-derived anion-dependent interaction scale and an electron donor ability scale (^23^Na NMR spectroscopy of Na^+^ dissolved in different ILs).^68^ There are far more ILs available now, so attempts at finding linear correlations can be more robust. There are a number of interaction scales available in the literature for the strength of anion–cation interactions, e.g., ^1^H NMR spectroscopy of C^2^–H of [C_4_C_1_Im]^+^ in a molecular solvent,^69,70^ an electron donor scale from UV–vis spectroscopy of a Cu^II^ cationic complex dissolved in different ILs,^70^ and ion pair calculations of hydrogen-bond strength,^71^ and also for the strength of anion–neutral molecule interactions (more often called Lewis basicity/hydrogen-bond acceptor ability/electron donor number).^69,72−78^ It is currently unclear whether these scales capture the same interactions as the XPS-derived interaction scale.

In this study, we intend to primarily answer four questions. (i) Where do key anions, e.g., [SCN]^−^ and [HSO_4_]^−^, come on the XPS-derived interaction strength scale? (ii) Is the XPS-derived anion-dependent interaction strength scale due to initial-state (i.e., ground-state) effects or final-state effects? (iii) What is the best explanation for the XPS-derived anion-dependent interaction scale, e.g., occupied valence state/unoccupied valence state interaction, polarizability, or electrostatic interactions? (iv) Does the experimental XPS-derived interaction strength scale correlate with other measures of the strength of anion–cation interactions or the strength of anion–neutral molecule interactions? We answered these questions using a combination of core XPS, valence XPS, and AIMD plus DFT to obtain realistic structures of ILs, followed by single-point DFT calculations to obtain *E*_B_(core,calc.).

## 2 Methods

### 2.1 Ionic Liquid Synthesis

Details of IL synthesis/purchase for the six ILs included here where core XPS was previously unpublished ([C_8_C_1_Im]_2_[Bi_2_Cl_8_], [C_8_C_1_Im][CF_3_CO_2_], [C_8_C_1_Im][SnBr_3_], [C_8_C_1_Im]_2_[Zn_3_Cl_8_], [C_8_C_1_Im][FSI] where [FSI]^−^ = bis(fluorosulfonyl)imide, [C_8_C_1_Im][InBr_4_]) are given in Section 1 in the ESI. The synthetic procedure for [C_8_C_1_Im]_2_[Zn_3_Cl_8_] was also given in ref (79).

### 2.2 X-ray Photoelectron Spectroscopy

Laboratory-based XPS was recorded for six ILs ([C_8_C_1_Im]_2_[Bi_2_Cl_8_], [C_8_C_1_Im][CF_3_CO_2_], [C_8_C_1_Im][SnBr_3_], [C_8_C_1_Im]_2_[Zn_3_Cl_8_], [C_8_C_1_Im][FSI], [C_8_C_1_Im][InBr_4_]) at the University of Reading on a Thermo Scientific ESCALAB 250 monochromated Al Kα source (*hν* = 1486.6 eV) spectrometer. A drop of IL was placed directly onto a stainless steel sample plate. This sample was placed in a loadlock, and the pressure was reduced to 10^–7^ mbar by pumping down for >6 h. After attaining the required pressure, the IL was transferred to the analysis chamber. Etching was carried out using a rastered 500 eV Ar^+^ ion beam (20 s for [C_8_C_1_Im]_2_[Bi_2_Cl_8_] and 500 s for [C_8_C_1_Im]_2_[Zn_3_Cl_8_]). For both [C_8_C_1_Im][CF_3_CO_2_] and [C_8_C_1_Im]_2_[Zn_3_Cl_8_], an area scan was performed to minimize sample charging/damage. Acquisition parameters were matched to give comparable energy resolution with data already published; a pass energy of 20 eV was used for core-levels.

All experimental XP spectra were fitted using CasaXPS software. Fitting was carried out using a Shirley background and GL30 line shapes (70% Gaussian, 30% Lorentzian). Peak constraints used are outlined in Section 2 in the ESI. Relative sensitivity factors from ref (80) were used to ensure the experimental stoichiometries matched the nominal stoichiometries.

All XPS *E*_B_(core,exp.) values were shifted relative to *E*_B_(C_alkyl_ 1s,exp.) = constant value, chosen as *E*_B_(C_alkyl_ 1s,exp.) = 285.00 eV here, as standard for ILs;^15,30,81^ more details on charge referencing experimental XPS of ILs are given in Section 2 in the ESI. From multiple measurements of the same IL, we have found that the uncertainty in *E*_B_(N_cation_ 1s,exp.) is smaller than given in references published in 2010 and 2011;^15,30^ here, we have used ±0.05 eV for uncertainty in *E*_B_(N_cation_ 1s,exp.).

**Figure 2:**
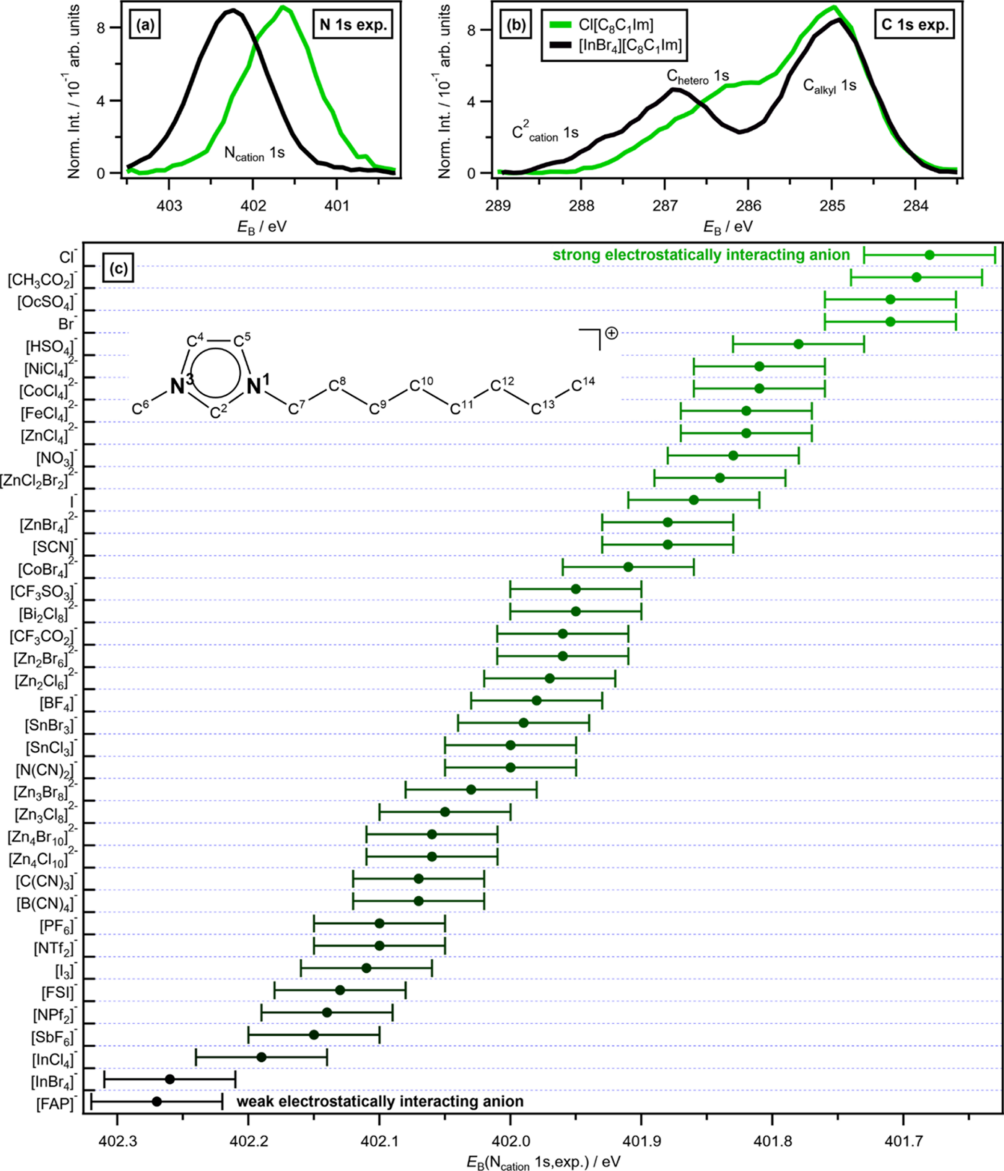
Experimental XPS for [C_8_C_1_Im][A] where [A]^−^ = Cl^–^ and [InBr_4_]^−^: (a) experimental N 1s XPS; (b) experimental C 1s XPS. (c) *E*_B_(N_cation_ 1s,exp.) for 39 different anions measured for [C_*n*_C_1_Im][A] (see Table S5 in the ESI for the numerical values). The estimated uncertainty is ±0.05 eV. Data from this paper apart from [NO_3_]^−^,^15^ [NPf_2_]^−^,^15^ [CH_3_CO_2_]^−^,^33^ [SbF_6_]^−^,^36^ and [I_3_]^−^.^18^ Large *E*_B_(N_cation_ 1s,exp.) = weak electrostatically interacting anion (black) and small *E*_B_(N_cation_ 1s,exp.) = strong electrostatically interacting anion (green). Inset in (c): structure of the cation 1-octyl-3-methylimidazolium, [C_8_C_1_Im]^+^, with two N_cation_ atoms highlighted.

*E*_B_(N_cation_ 1s,exp.) for [C_8_C_1_Im]^+^-based ILs are used as the primary measure of the anion-dependent interaction strength (e.g., Figure 2a,c). *E*_B_(C_hetero_ 1s,exp.) and *E*_B_(C^2^ 1s,exp.) can also be used as a secondary measure of the anion-dependent interaction strength (Figure 2b), although *E*_B_(N_cation_ 1s,exp.) is usually used, as C_anion_ 1s peaks can overlap with C_hetero_ 1s and C^2^ 1s peaks, e.g., for [SCN]^−^-based ILs,^66^ making fitting more challenging when obtaining *E*_B_(C_hetero_ 1s,exp.) and *E*_B_(C^2^ 1s,exp.) than *E*_B_(N_cation_ 1s,exp.).

A major difference between the measurement conditions for the XPS-derived interaction scale and the other eight interaction scales discussed above is that the IL XPS measurements are made under ultrahigh vacuum (UHV) conditions. These UHV conditions mean that residual molecular solvents will have vaporized prior to XPS measurements, giving ultrapure samples from a molecular solvent contamination perspective. Furthermore, the element-specific nature of XPS means that a number of ionic impurities, e.g., Na^+^, can be observed. Therefore, we can have very high confidence in the purity of our ILs for the XPS measurements.

### 2.3 Ab Initio Molecular Dynamics

The ILs [C_8_C_1_Im][SCN], [C_8_C_1_Im][NTf_2_], and [C_8_C_1_Im]Cl were each simulated using a 32-ion pair model with densities and temperatures, as listed in Table 1. The AIMD was calculated with the Quickstep code in CP2K, from the Gaussian and plane wave method (GPW) and using the direct inversion in iterative subspace (DIIS) technique. Pre-equilibration was performed using the classical force field DREIDING, and then the AIMD simulation was run at a time step of 1 fs for 30 ps. All simulations were controlled by a Nosé thermostat in the canonical NVT ensemble. The Perdew–Burke–Ernzerhof (PBE) functional^82^ was applied, with Grimme’s D2 corrections^83,84^ to account for dispersion interactions. An increased simulation temperature was used (Table 1) to reduce the viscosity in the system and allow for equilibrium to be achieved faster, thus reducing the computational cost of the calculation while preventing thermal decomposition.

**Table 1:** Temperature and Density Used for Each Ionic Liquid

ionic liquid	temperature/K	density/g cm^–3^
[C_8_C_1_Im][SCN]	398	0.89
[C_8_C_1_Im][NTf_2_]	398	1.32
[C_8_C_1_Im]Cl	498	1.01

### 2.4 Core-Level Binding Energy and Electrostatic Site Potential Calculations

Calculations of the *E*_B_(core,calc.) were performed using the Vienna Ab initio Simulation Package (VASP).^85^ Three configurations of the average energies calculated in AIMD for each IL were chosen to calculate the *E*_B_ values across the 96-ion pairs (3 × 32-ion pairs). The PBE exchange-correlation functional was employed, and the core–valence electron interactions were described using the projector-augmented wave (PAW) potentials.^86,87^ The kinetic energy cutoff in the plane wave basis set expansion was set to 400 eV for all ILs. All core-level energies were calculated using the initial-state approximation.

**Figure 3:**
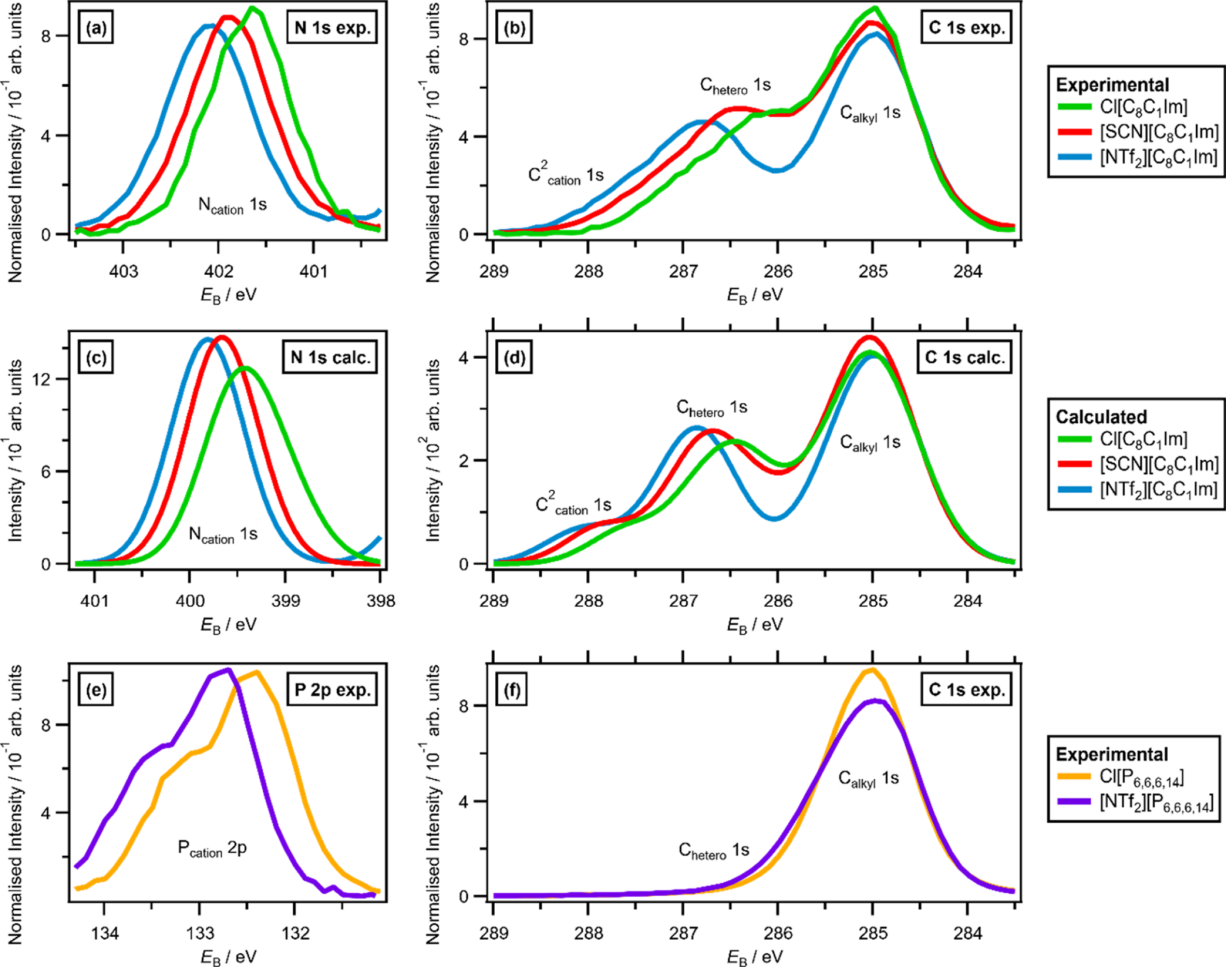
Experimental and calculated XPS for [C_8_C_1_Im][A], where [A]^−^ = Cl^–^, [SCN]^−^, and [NTf_2_]^−^: (a) experimental N 1s XPS, (b) experimental C 1s XPS, (c) calculated N 1s XPS for three configurations of each IL (fwhm = 0.7 eV), and (d) calculated C 1s XPS for three configurations of each IL (fwhm = 0.7 eV). Experimental XPS for [P_6,6,6,14_][A],where [A]^−^ = Cl^–^ and [NTf_2_]^−^: (e) experimental P 2p XPS and (f) experimental C 1s XPS.

**Figure 4:**
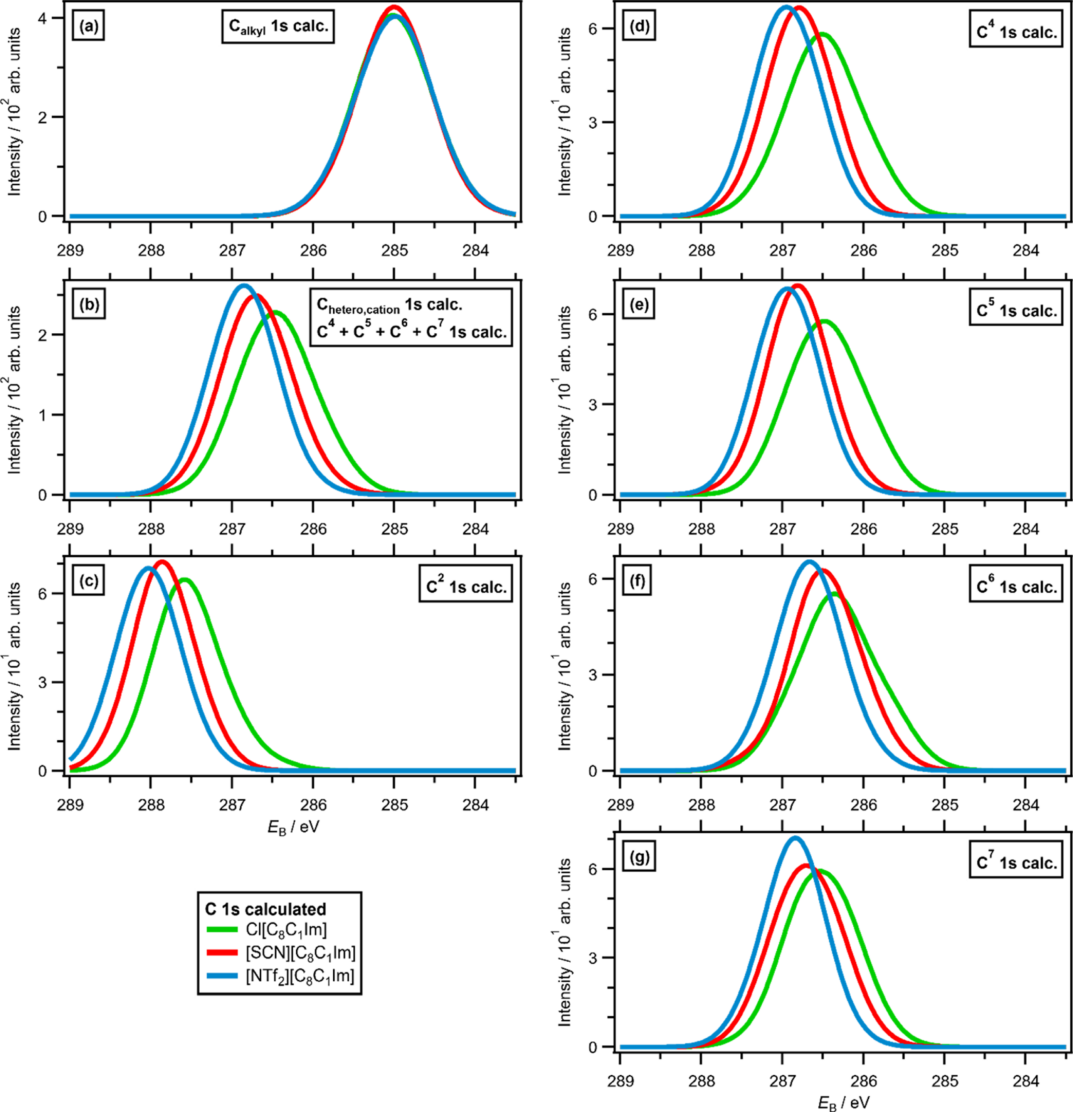
Calculated C 1s XPS for [C_8_C_1_Im][A], where [A]^−^ = Cl^–^, [SCN]^−^, and [NTf_2_]^−^ for three configurations of each IL (fwhm = 0.7 eV): (a) C_alkyl_ 1s XPS, (b) C_hetero_ 1s XPS, (c) C^2^ 1s XPS, (d) C^4^ 1s XPS, (e) C^5^ 1s XPS, (f) C^6^ 1s XPS, and (g) C^7^ 1s XPS.

To produce the calculated XP spectra (Figures 3 and 4), a Gaussian–Lorentzian Product (GLP) function was applied to each calculated *E*_B_ data point for each core-level using eq 1 and then summed to produce calculated XPS data. The mixing parameter, *m*, was set to 0.3, as in line with experimental peak fitting, and the function width, *F*, was set to 0.7 eV.
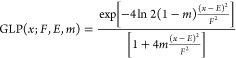
1

**Figure 5:**
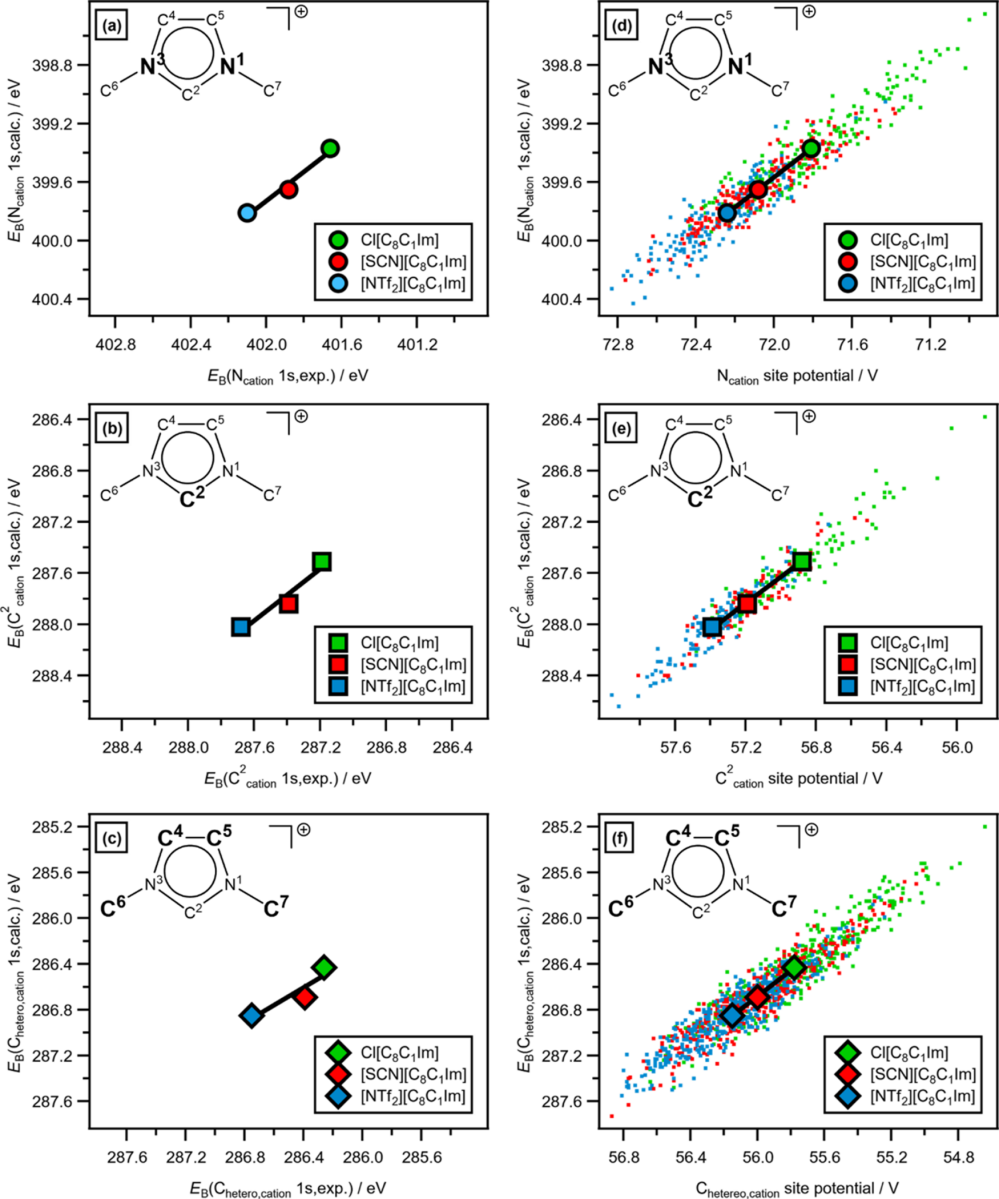
Experimental and calculated XPS *E*_B_ and site potential data for [C_8_C_1_Im][A], where [A]^−^ = Cl^–^, [SCN]^−^, and [NTf_2_]^−^: (a) average *E*_B_(N_cation_ 1s,calc.) (for three configurations of each IL) versus *E*_B_(N_cation_ 1s,exp.), (b) average *E*_B_(C^2^_cation_ 1s,calc.) (for three configurations of each IL) versus *E*_B_(C^2^_cation_ 1s,exp.), and (c) average *E*_B_(C_hetero_ 1s,calc.) (for three configurations of each IL) versus *E*_B_(C_hetero_ 1s,exp.). Calculated XPS *E*_B_ and site potential data (both all individual atoms and the average for three configurations of each IL): (d) *E*_B_(N_cation_ 1s,calc.) versus N_cation_ site potential, (e) *E*_B_(C^2^ 1s,calc.) versus C^2^ site potential, and (f) *E*_B_(C_hetero_ 1s,calc.) versus C_hetero_ site potential.

To produce the calculated *E*_B_ used in Figure 5, an average was taken of the relevant atoms for all three configurations of each IL, i.e., 3 × 32 × *n E*_B_ values, where *n* reflects the different number of atoms for each grouping (e.g., *n* = 2 for N_cation_ for each IL ion pair).

Electrostatic site potentials were taken from VASP version 6.4.1, where the average of the electrostatic potential is taken from the core of an atom at a given position. To produce the average calculated electrostatic site potentials used in Figure 5d–f, e.g., the N_cation_ electrostatic site potential for each IL, the same averaging method was used as for *E*_B_.

### 2.5 Aligning Calculated XPS and Electrostatic Site Potential Data

Both *E*_B_(core,calc.) and calculated electrostatic site potentials need to be aligned to allow comparison, as the nature of the AIMD plus DFT bulk IL calculation method used here does not give a simple reference, e.g., there is no vacuum level.

To allow comparisons of our calculated XPS data to our experimental core XPS data, calculated XPS data were aligned with our chosen internal energy reference, *E*_B_(C_alkyl_ 1s,calc.) = 285.00 eV. The average *E*_B_(C_alkyl_ 1s,calc.) value for each IL was shifted to match 285.00 eV for calculated XP spectra, the average *E*_B_(core,calc.) values, and the 3 × 32 × *n E*_B_(core,calc.) values; *E*_B_(core,calc.) for other core-levels (e.g., N 1s) were shifted the same for that particular IL. Hence, only relative *E*_B_ values are meaningful; absolute values are not considered here, only the difference between binding energies, Δ*E*_B_. Therefore, *E*_B_(C_alkyl_ 1s,exp.) and *E*_B_(C_alkyl_ 1s,calc.) (Figures 2b, 3b,d,f, and 4a) match perfectly, as they are charge referenced to match at *E*_B_(C_alkyl_ 1s) = 285.00 eV.

The average calculated electrostatic site potentials were set to a common reference, chosen as C_alkyl_ electrostatic site potential = 54.26 V for the average C_alkyl_ site potential of C^8^ to C^14^ for [C_8_C_1_Im][SCN] (Figure 1a for the structure).

## 3 Results and Discussion

### 3.1 XPS-Derived Anion-Dependent Interaction Strength Scale for 39 Different Anions

An XPS-derived anion-dependent interaction strength scale for 39 different anions is presented here (Figure 2c),^15,16,18,33^ with at least 18 anions placed on the scale for the first time, e.g., the weakly interacting [FSI]^−^ anion, which is very important for batteries.^88^ A major advantage of this XPS-derived scale is the ability to measure both ILs that are inherently colored (a problem for UV–vis spectroscopy measurements, e.g., [Bi_2_Cl_8_]^2–^) and ILs that are magnetic (a problem for NMR spectroscopy, e.g., [CoBr_4_]^2–^). Two examples of placing anions on the XPS-derived interaction strength scale are given in Figure 2a,b: Cl^–^ and [InBr_4_]^−^. Cl^–^ has been measured previously using XPS and placed on the interaction scale as among the most strongly interacting anions for ILs (Figure 2c).^15^ XPS data are published here that allow the placement of the key cyano-based anions on the XPS-derived interaction scale for the first time. Other new anions include [HSO_4_]^−^ and halometallate anions, which span a wide range of anion interaction strengths from strong, e.g., [MCl_4_]^2–^ (where M = Co^2+^, Ni^2+^, Fe^2+^, Zn^2+^), to weak, e.g., [InCl_4_]^−^ and [InBr_4_]^−^ (Figure 2c); [FAP]^−^ (tris(pentafluoroethyl)trifluorophosphate) is the most weakly interacting anion measured using XPS but is the same as [InBr_4_]^−^ within the uncertainty (Figure 2c). Perhaps somewhat surprisingly, the magnitude of the anion charge is not a major determinant in the anion interaction strength; doubly charged anions are not always strongly interacting (e.g., [Zn_4_Cl_10_]^2–^; Figure 2c^89^), while singly charged anions are not always weakly interacting (e.g., Cl^–^ and [CH_3_CO_2_]^−^ (acetate); Figure 2c).

The maximum Δ*E*_B_(N_cation_ 1s,exp.) for [C_8_C_1_Im][A] with different [A]^−^ was 0.59 eV (402.27 eV for [FAP]^−^ to 401.68 eV for Cl^–^; Figure 2c). This Δ*E*_B_(N_cation_ 1s,exp.) value is very small compared to the largest Δ*E*_B_(N 1s,exp.) in our XPS data set for all ILs, Δ*E*_B_(N_anion_ 1s,exp.) = 8.66 eV for [NO_3_]^−^ versus [SCN]^−^ (caused by the difference in covalent bonding in these anions; Figure S13 in the ESI). Moreover, Δ*E*_B_(N_cation_ 1s,exp.) was 0.80 eV for [C_8_C_1_Im]^+^ versus [N_4,1,1,1_]^+^. These and other comparisons (Figure S13 in the ESI) highlight that the change in Δ*E*_B_(N_cation_ 1s,exp.) for [C_8_C_1_Im][A] with different [A]^−^ is smaller than changes caused by differences in covalent bonding in individual ions.

### 3.2 XPS-Derived Anion-Dependent Interactions: Ground-State Explanation

Our AIMD plus (ground-state) DFT calculations can be validated against our experimental XPS data. First, intramolecular evidence: for XPS of all [C_*n*_C_1_Im]^+^-based ILs, the *E*_B_ order of *E*_B_(C^2^ 1s) > *E*_B_(C_hetero_ 1s) > *E*_B_(C_alkyl_ 1s) from both experimental and calculated XPS (Figures 2b and 3b,d) matches to the electronegativity of the atom covalently bonded to the carbon atom in question, i.e., C^2^ has two C–N bonds, all C_hetero_ have one C–N bond, and C_alkyl_ has no C–N bonds (only C–C and C–H). This observation is exactly as expected based on the XPS literature.^25,26,90,91^ Second, interion evidence: anion–cation interion interactions between the [C_8_C_1_Im]^+^ cation and the anions are captured. For [C_8_C_1_Im][NTf_2_] (both N 1s and C 1s) and [C_8_C_1_Im][SCN] (N 1s) (both N 1s and C 1s for [C_4_C_1_Im][SCN] in ref (66)), *E*_B_(anion core,exp.) minus *E*_B_(cation core,exp.) matches very well to *E*_B_(anion core,calc.) minus *E*_B_(cation core,calc.) (Figure S14 in the ESI).

There are excellent matches between the experimental and calculated XPS for cation-based contributions (Figures 3a–d and 5a–5c) for [C_8_C_1_Im][A] (where [A]^−^ = [NTf_2_]^−^, [SCN]^−^, Cl^–^). For N_cation_ 1s XPS, there is an excellent match of the experimental and calculated N_cation_ 1s XPS (Figure 3a,c respectively) for [C_8_C_1_Im][A] (where [A]^−^ = [NTf_2_]^−^, [SCN]^−^, Cl^–^). Both experiments and calculations find the same order for *E*_B_(N_cation_ 1s,exp.) of [NTf_2_]^−^ > [SCN]^−^ > Cl^–^ (see Figure 2c for comparisons to values for other anions). A comparison of the experimental versus calculated C^2^_cation_ 1s XPS (Figure 3b,d, respectively) and experimental versus calculated C_hetero_ 1s XPS (Figure 3b,d, respectively) for [C_8_C_1_Im][A] (where [A]^−^ = [NTf_2_]^−^, [SCN]^−^, Cl^–^) show the same order of [NTf_2_]^−^ > [SCN]^−^ > Cl^–^, matching the observed order for *E*_B_(N_cation_ 1s,exp.). Breakdowns of C_hetero_ 1s and C^2^ 1s for [NTf_2_]^−^ versus [SCN]^−^ versus Cl^–^ also highlight these trends (Figure 4b,c, respectively). Analysis is slightly more complicated for the [SCN]^−^ anion for C 1s than N_cation_ 1s because the carbon from the [SCN]^−^ anion (C_anion_ 1s) contributes in a similar *E*_B_ region to C_hetero_ 1s and C_alkyl_ 1s (see experimental evidence for this finding in ref (66)); this C_anion_ 1s contribution can be easily separated in the calculated XPS but is more challenging to account for when fitting the experimental XPS.

The magnitude of the *E*_B_ shifts in *E*_B_(N_cation_ 1s), *E*_B_(C^2^_cation_ 1s), and *E*_B_(C_hetero_ 1s) for [C_8_C_1_Im][A] (where [NTf_2_]^−^, [SCN]^−^, and Cl^–^) matches well for experimental versus calculated XPS both for the XP spectra (Figure 3a,c, respectively) and for *E*_B_(core) values, with excellent linear correlations of three data points for the average *E*_B_(N_cation_ 1s,calc.) versus *E*_B_(N_cation_ 1s,exp.) (Figure 5a), and the same plots for *E*_B_(C^2^_cation_ 1s) and *E*_B_(C_hetero_ 1s) (Figure 5b,c, respectively).

These observations show that the XPS-derived anion-dependent interaction strength scale for ILs is a ground-state effect (in XPS language, an initial-state effect) and not a product of electron density redistribution after the core-hole is created in XPS (i.e., not an XPS final-state effect). This finding strongly backs the assumption used regularly in the IL XPS literature that initial-state effects dominate *E*_B_ shifts for ILs.^15−22,27−29,31−64^ Therefore, the effect of different anions on the cation can be related to ground-state differences.

### 3.3 XPS-Derived Anion-Dependent Interactions: An Electrostatic Explanation for the Cation Changes

The average *E*_B_(core,calc.) linearly correlates with the average calculated electrostatic site potentials, i.e., *E*_B_(N_cation_ 1s,calc.) versus N_cation_ electrostatic site potential (Figure 5b), *E*_B_(C^2^_cation_ 1s,calc.) versus C^2^_cation_ electrostatic site potential (Figure 5d), and *E*_B_(C_hetero,cation_ 1s,calc.) versus C_hetero_ electrostatic site potential (Figure 5f). Furthermore, *E*_B_(core,exp.) linearly correlates with the average calculated electrostatic site potentials for C^2^_cation_, N_cation_, and C_hetero_ (Figure S16 in the ESI). For each IL, when all calculated data points are considered rather than the average calculated values, excellent linear correlations are found (for N_cation_, C^2^_cation_, and C_hetero_ in Figure 5d,e,f, respectively), e.g., for [C_8_C_1_Im][SCN], when all 192 relevant atoms from three configurations are considered, calculated *E*_B_(N_cation_ 1s,calc.) shows an excellent linear correlation with the N_cation_ electrostatic site potential (Figure 5d). Therefore, it can be concluded that the electrostatic site potentials for all three of the imidazolium ring carbon atoms (C^2^, C^4^, and C^5^), the two imidazolium ring nitrogen atoms (N^1^ and N^3^) and the two N–CH_2_R carbon atoms (C^6^ and C^7^) are affected by the anion identity in the order [NTf_2_]^−^ > [SCN]^−^ > Cl^–^. This observation that all of these imidazolium-based atoms are affected the same by the different anions is evidence of the dominant role of electrostatic interactions and relative unimportance of occupied valence state/unoccupied valence state interactions, as for interactions involving specific valence states one would expect some atoms, especially the ring atoms, to be affected more than other atoms.

**Figure 6:**
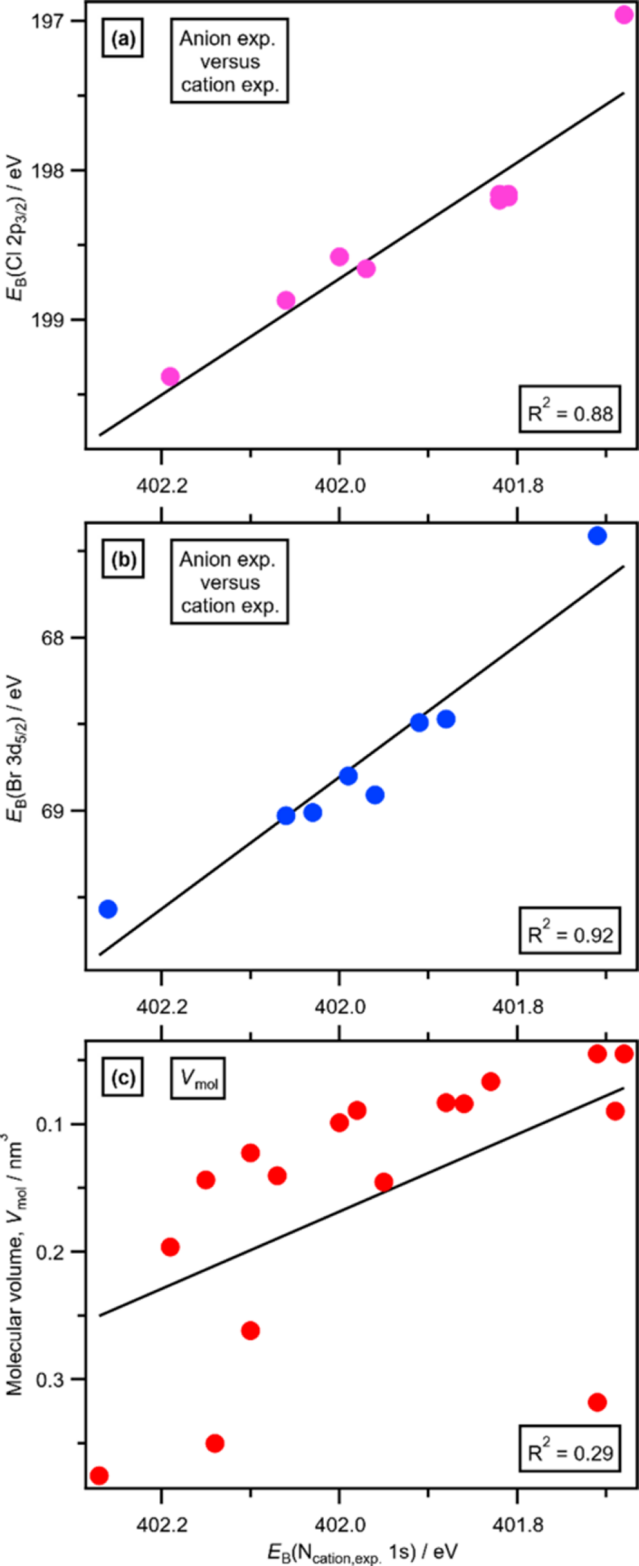
Anion properties plotted against *E*_B_(N_cation_ 1s,exp.): (a) *E*_B_(Cl 2p_3/2_,exp.) versus *E*_B_(N_cation_ 1s,exp.) for nine Cl-containing ILs, (b) *E*_B_(Br 3d_5/2_,exp.) versus *E*_B_(N_cation_ 1s,exp.) for eight Br-containing ILs, and (c) calculated anion molecular volume, *V*_mol_, taken from ref (93) versus *E*_B_(N_cation_ 1s,exp.) for 17 ILs (Table S5 in the ESI for values used).

There is an excellent linear correlation between the anion core-level *E*_B_(Cl 2p_3/2_,exp.) and *E*_B_(N_cation_ 1s,exp.) for eight Cl-containing ILs (Figure 6a). Furthermore, there is also an excellent linear correlation between the anion core-level *E*_B_(Br 3d_5/2_,exp.) and *E*_B_(N_cation_ 1s,exp.) for eight Br-containing ILs (Figure 6b). The *R*^2^ values for both of these linear correlations would be increased by removing the free halide data points (i.e., Cl^–^ and Br^–^ anions), potentially due to a different interaction mechanism with the cations for the free halides compared to the halometallate anions. The experimental anion core-level *E*_B_(anion core) can be taken as a measure of the electrostatic site potential at these anion atoms. Therefore, these linear correlations demonstrate that the anion-dependent interaction strength for the Cl- and Br-containing ILs can be explained by electrostatic interactions between the halide atom(s) and both the imidazolium ring and the two N–CH_2_R carbon atoms of the cation. Furthermore, a plot of *E*_B_(O_anion_ 1s,exp.) versus *E*_B_(N_cation_ 1s,exp.) for eight O-containing anions gave a reasonable linear correlation (Figure S18 in the ESI), backing up our arguments made for the Cl- and Br-containing anion data sets.

Beyond the multiple linear correlations between *E*_B_ and electrostatic site potential, there are three pieces of evidence to support the finding that electrostatic interactions explain the anion-dependent interactions (note that a fourth piece of evidence is given in Section 3.4). First, calculations show that the average *E*_B_(C 1s,calc.) values for all four individual carbon atoms that contribute to *E*_B_(C_hetero_ 1s,calc.), i.e., *E*_B_(C^4^ 1s,calc.), *E*_B_(C^5^ 1s,calc.), *E*_B_(C^6^ 1s,calc.), and *E*_B_(C^7^ 1s,calc.), match the *E*_B_(C_hetero_ 1s,exp.) trend, i.e., [NTf_2_]^−^ > [SCN]^−^ > Cl^–^ (Figure 4d–4g). The lowest unoccupied molecular orbital (LUMO) for [C_4_C_1_Im]^+^ was found by calculations to be antibonding π-type located on the imidazolium ring,^92^ i.e., on the imidazolium ring carbon (C^2^, C^4^, C^5^) and nitrogen atoms. Therefore, if electron donation occurred from a specific anion occupied valence state to a specific cation unoccupied valence state, one would expect the imidazolium ring carbon (C^2^, C^4^, C^5^) and nitrogen (N^1^, N^3^) atoms to be more affected than the N–CH_3_ (C^6^) and N–CH_2_–CH_2_– (C^7^) carbon atoms, which is not the case. This finding suggests that the XPS-derived anion-dependent interactions for ILs are best explained by electrostatic interactions rather than an anion occupied valence state to a cation unoccupied valence state interaction. Second, cationic *E*_B_ all have the same order for these different [A]^−^, irrespective of the cation identity.^15,16,19−22^ These organic cations have both aromatic ([C_8_C_1_Im]^+^, [C_*n*_C_1_C_1_Im]^+^, [C_8_Py]^+^) and alkyl ([P_6,6,6,14_]^+^, [C_8_C_1_Pyrr]^+^, tetraalkylammonium) headgroups; if electron donation occurred from a specific anion-occupied valence state to a specific cation-unoccupied valence state, one would expect the identity of the cation and therefore the identity of the cation-unoccupied valence state to be a strong factor. Third, as reported in ref (15), the results for the nonmethylated ([C_8_C_1_Im]^+^) versus methylated ([C_8_C_1_C_1_Im]^+^) imidazolium cations with [NTf_2_]^−^ and Br^–^ anions rule out (atom-specific electrostatic) hydrogen bonding interactions to explain any trends. Note that these electrostatic interactions are not constrained to any pair of atoms, unlike bonding or hydrogen bonding interactions - which are for specific atom pairs - but result from contributions from all of the atoms in the vicinity of a given site.

The calculated anion size, which is captured by the calculated anion molecular volume (*V*_mol_) taken from ref (93), did not linearly correlate with experimental *E*_B_(N_cation_ 1s,exp.) for 17 ILs (Figure 6c). Data for the experimental size of the IL, the molecular volume of one IL ion pair, also did not linearly correlate with experimental *E*_B_(N_cation_ 1s,exp.) for 10 ILs.^15^ In ref (15), a linear correlation was noted for six of the smaller, more strongly interacting ILs. However, such a linear correlation is very much not seen in our data, given the inclusion of [OcSO_4_]^−^, which is one of the largest anions (Figure 6c) but one of the strongest interacting anions on our XPS-derived scale (Figure 1c). [OcSO_4_]^−^ is found to be a midrange to strongly interacting anion on other anion interaction strength scales.^68−71,74^ A linear correlation was found for eight ILs between anion interaction strength (i.e., donor number) and molar concentration (proportional to the inverse of the IL size).^94^ Again, the inclusion of [OcSO_4_]^−^ in the data set in ref (94) would lead to no linear correlation. Therefore, it can be concluded that anion size is a weak factor in determining anion–cation interaction strengths, and factors such as the identity and number of coordinating atoms will be more important.

There is not a linear correlation between the XPS-derived anion-dependent interaction strength scale and anion polarizability, which further supports our finding that the anion-dependent interaction scale has an electrostatic explanation. Most importantly, *E*_B_(N_cation_ 1s,exp.) for [C_8_C_1_Im]Cl is smaller than that for [C_8_C_1_Im]I,^15^ showing that Cl^–^ interacts more strongly with [C_8_C_1_Im]^+^ than I^–^, matching to multiple other interaction strength scales;^68,69,74,76−78^ however, the polarizability of I^–^ is far larger than that of Cl^–^.^95^ As well as anion polarizability, other data are available on polarizability for IL anions, e.g., individual atom polarizabilities^96^ and polarizability scaled to the size of the anion.^97^ [B(CN)_4_]^−^ is far more polarizable than Cl^–^ when anion size is taken into account,^97^ but *E*_B_(N_cation_ 1s,exp.) for [C_8_C_1_Im]Cl is smaller than that for [C_6_C_1_Im][B(CN)_4_], showing that [B(CN)_4_]^−^ interacts far more weakly with cations than Cl^–^ (Figure 2c). For [PF_6_]^−^ versus [NTf_2_]^−^, *E*_B_(N_cation_ 1s,exp.) values are the same,^15,30^ but the atomic polarizability for F in [PF_6_]^−^ is much smaller than the atomic polarizability for O in [NTf_2_]^−^.^96^ There is currently insufficient data in the literature on Cl- and Br-containing anions to judge whether atom polarizability for this subset of anions correlates with our anion-dependent interaction strength scale.

The data on *E*_B_ and electrostatic site potentials highlights an important point that is almost always overlooked when considering experimental XPS data for ILs. A specific atom type for a specific IL gives a relatively large range of calculated *E*_B_ and electrostatic site potential values, e.g., for [C_8_C_1_Im][SCN], *E*_B_(C^2^ 1s,calc.) ranges from 287.17 to 288.41 eV (32 C^2^ atoms in each simulation box, three configurations equals 96 C^2^ atoms), a difference of 1.24 eV (Figure 5h). While experimental XPS can capture average *E*_B_(core,exp.) from fitting and give an insight into the range of *E*_B_(core,exp.) from the peak width in the fitting, this insight from AIMD plus DFT highlights how in liquids, the localized electronic structure varies greatly across the liquid phase and is strongly dependent on the local environment/structure. Furthermore, the variation of 1.24 eV is far greater than the average *E*_B_ difference for *E*_B_(C^2^ 1s,calc.) of 0.51 eV caused by changing from [C_8_C_1_Im]Cl to [C_8_C_1_Im][NTf_2_]. These observations highlight that within even a relatively small number of ions in a simulation box, there is a great deal of range in the localized electronic structure. This variation is important to keep in mind when considering reactivity, which is likely to occur for unusual structures.

Overall, the anion-dependent interaction strength scale can be explained using a simple electrostatic model. Thus, the anion-dependent electrostatic interaction strength scale is a more appropriate name.

### 3.4 Electrostatic Interactions Explain Anion–Cation and Anion–Neutral Molecule Interactions

**Figure 7:**
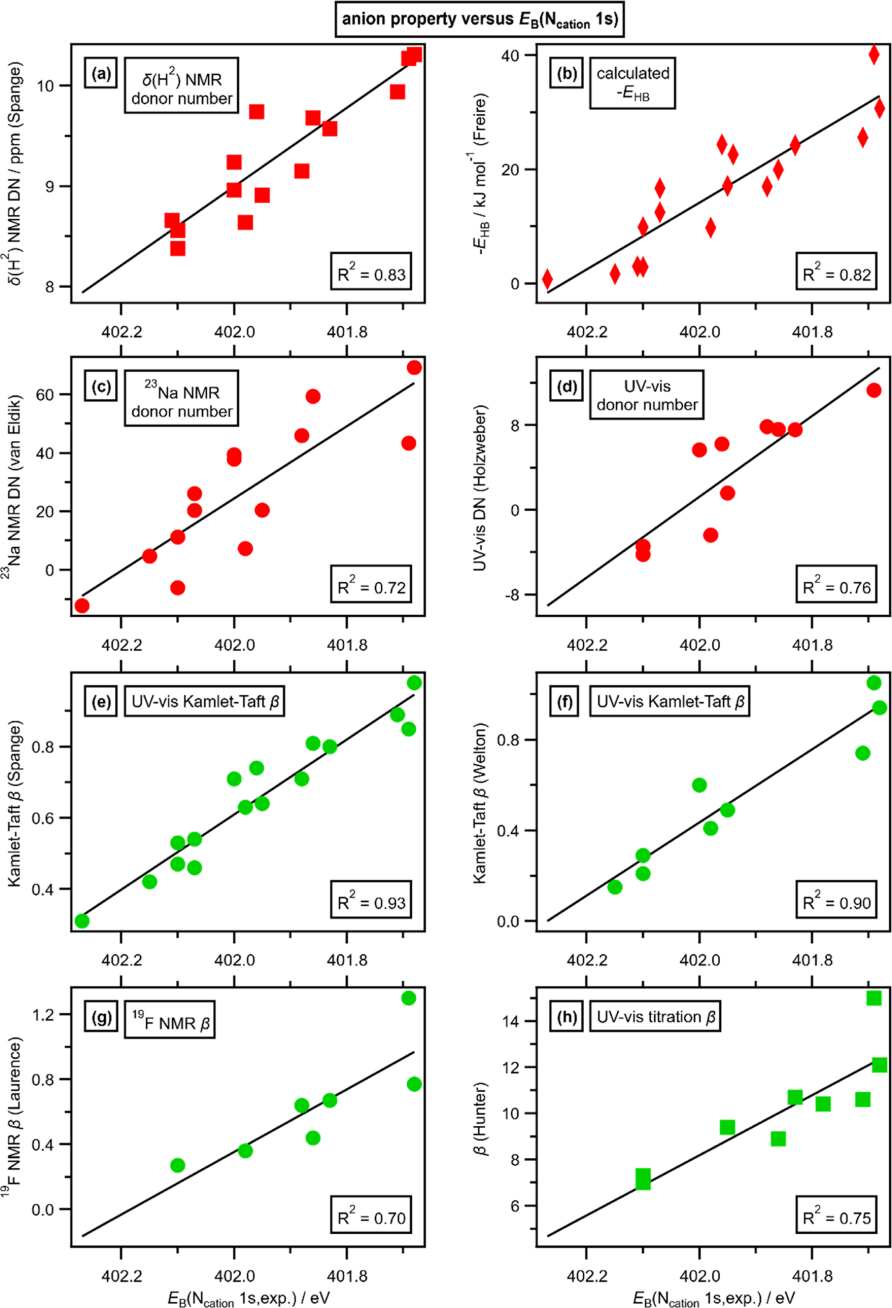
Measures of anion Lewis basicity/electron donor ability/hydrogen-bond acceptor ability plotted against *E*_B_(N_cation_ 1s,exp.): (a) electron donor number measured from the chemical shifts δ(H) of the C^2^–H proton by ^1^H NMR spectroscopy of [C_4_C_1_Im][A] in the molecular solvent CD_2_Cl_2_;^69,70^ (b) hydrogen-bond basicity calculated using ion pairs in COSMO-RS (COnductor-like Screening MOdel for Real Solvents);^71^ (c) anion electron donor numbers measured from the chemical shift δ(Na^+^) by ^23^Na NMR spectroscopy of Na[ClO_4_] dissolved in [C_4_C_1_Im][A] neat ionic liquids;^68^ (d) anion electron donor numbers measured from the peak shift of the copper complex [Cu(acetylacetonate)(tetramethylethylenediamine)][ClO_4_] by UV–vis spectroscopy in [C_4_C_1_Im][A] neat ionic liquids;^70^ (e) Kamlet–Taft hydrogen-bond acceptor numbers using UV–vis spectroscopy comparisons of two different neutral dye molecules in [C_4_C_1_Im][A] neat ionic liquids;^74^ (f) Kamlet–Taft hydrogen-bond acceptor numbers using UV–vis spectroscopy comparisons of two different neutral dye molecules in [C_4_C_1_Im][A] neat ionic liquids;^75^ (g) hydrogen-bond acceptor numbers measured from the chemical shift δ(F) by ^19^F NMR spectroscopy of a neutral fluorinated dye molecule dissolved in [C_4_C_1_Im][A] neat ionic liquids;^76^ and (h) hydrogen-bond acceptor values for anions measured by titration using UV–vis spectroscopy of three different neutral dye molecules in two different organic solvents (MeCN and CHCl_3_).^78^ Red = cation probes. Green = neutral probes. Circles = measured in neat ILs, the same as *E*_B_(N_cation_ 1s,exp.). Squares = measured diluted in a molecular solvent. Diamonds = calculations.

Our newly established anion-dependent electrostatic interaction strength scale correlates linearly to four different anion–cation interaction strength scales (Figure 7a–d): ^23^Na NMR spectroscopy of a Na^+^ cation,^68^ UV–vis spectroscopy of a Cu^II^-based cation,^70^ chemical shifts δ(H) of the C^2^–H proton for [C_4_C_1_Im]^+^ using ^1^H NMR spectroscopy,^69,70^ and hydrogen-bond basicity calculated using [C_4_C_1_Im][A] ion pairs in COSMO-RS (COnductor-like Screening MOdel for Real Solvents).^71^ Our anion–cation interaction strength scale also correlates linearly with four different anion–neutral molecule interaction strength scales, which are all measures of anion basicity or hydrogen-bond acceptor ability (Figure 7e–7h).

Two anions that are worth noting are Cl^–^ and [CH_3_CO_2_]^−^, which are generally the two strongest interacting anions. These two anions give the same interaction strength on our XPS scale (Figure 2c) but different values on other interaction strength scales (hydrogen-bond basicity (Figure 7b),^17^ donor number (Figure 7c),^18^ hydrogen-bond acceptor number (Figure 7g),^21^ hydrogen-bond acceptor value (Figure 7h).^22^) [CH_3_CO_2_]^−^ is the stronger interacting anion on three scales,^18,21,22^ and Cl^–^ is the stronger interacting anion on one scale.^18^ These differences are worthy of further investigation.

The linear correlations (Figure 7) strongly indicate that all eight of the anion–probe interaction strength scales are dominated by electrostatic interactions, as our XPS-derived scale is controlled by electrostatic interactions. The anion-dependent interaction strength scale is very likely independent of the probe identity, including both cations and neutral molecules as the probe. This observation provides a fourth piece of evidence to support the finding that electrostatic interactions explain the anion-dependent interactions (pieces of evidence one to three are given in Section 3.3). Furthermore, these linear correlations indicate that all nine of the anion-dependent interaction strength scales considered here are determined by properties of the anion only, i.e., intrinsic properties of the anion. At this stage, we do not have a single anion property, whether experimental or calculated, that captures the strength of the anion-dependent interaction scales, e.g., anion size does not work (Figure 6c). We expect the best chance of finding such an anion property will be through calculations.

## 4 Conclusions

Using XPS and AIMD plus DFT, we have gained significant new insights into anion–cation interactions. We have found evidence that the XPS-derived anion-dependent interaction scale is best rationalized by electrostatic interactions and not occupied valence state/unoccupied valence state interactions or polarizability-driven interactions. Hence, we now call this XPS-derived scale an anion-dependent electrostatic interaction strength scale.

The XPS-derived anion-dependent electrostatic interaction strength scale was due to initial-state (i.e., ground-state) effects and not final-state effects. Therefore, the conclusions drawn in the IL XPS literature, based on the assumption that initial-state effects dominate, are likely to be reliable.

Linear correlations were found between the anion-dependent electrostatic interaction strength scale and many other anion-dependent interaction strength scales, including scales measured using IL cations other than imidazolium, inorganic cations, and neutral molecules. These linear correlations strongly suggest that first, the anion–probe interactions are all primarily electrostatic; second, our electrostatic interaction strength scale captures some inherent, intrinsic property of anions, independent of the probe used to measure the interaction strength scale. These cationic and neutral probes are expected to have very different unoccupied valence states; therefore, the similarity of the trends observed for different anions adds further strength to our finding that electrostatic interactions are the key.

We have placed at least 18 anions on the experimental anion-dependent electrostatic interaction scale for the first time, giving an experimental scale made up of 39 anions, including [SCN]^−^, [C(CN)_3_]^−^, [B(CN)_4_]^−^, and [HSO_4_]^−^. [InBr_4_]^−^ is, along with [FAP]^−^ and [InCl_4_]^−^, the most weakly interacting anion on our scale, while Cl^–^, Br^–^, and [CH_3_CO_2_]^−^ are the most strongly interacting anions. We judge the effect of the different anions on the cation potential as not huge, smaller than most differences caused by varying covalent bonding in ions.

One recommendation from our results is that if one is using charge scaling to obtain atomic charges for use in MD simulations,^67,98,99^ one must consider anion-dependent charge scaling instead of a fixed value of charge scaling that is usually used. Furthermore, our experimental data set should prove excellent for validating calculations, whether that is for DFT of ion pairs/similar scale calculations, MD-DFT, or AIMD plus DFT. Our data set can greatly help answer a key question for such calculations, i.e., “do these calculations capture the anion-dependent interactions correctly?”

## Data Availability

The data underlying this study are openly available in the University of Reading Research Data Archive at https://doi.org/10.17864/1947.001317.

## Supporting Information Available

Ionic liquids studied and synthesis; peak fitting core level XP spectra and charge referencing; demonstrating purity; anion-cation interaction strength scale for 39 different anions; experimental versus calculated core XPS; linear correlations of *E*_B_ and ESP; proving that size does not matter strongly to anion interaction strength; *E*_B_(O_anion_ 1s) versus *E*_B_(N_cation_ 1s); [A]^−^-dependent [A]^-^-[C]^+^ interactions’ anion-cation and anion-neutral molecule interactions (PDF)
