# Supplementary material for: Anion-Dependent Strength Scale of Interactions in Ionic Liquids from X-ray Photoelectron Spectroscopy, Ab Initio Molecular Dynamics, and Density Functional Theory
Source: J Phys Chem B. 2024 May 10;128(20):5030–43. doi: 10.1021/acs.jpcb.4c00362 (PMC11129296; doi:10.1021/acs.jpcb.4c00362)
Supplement: Supplementary file 1 — jp4c00362_si_001.pdf [file jp4c00362_si_001.pdf]

Supporting Information

Anion-dependent strength scale of interactions in ionic liquids from X-ray photoelectron spectroscopy, *ab initio* molecular dynamics and density functional theory

Ekaterina Gousseva,[1] Frances K. Towers Tompkins,[1] Jake M. Seymour,[1] Lewis G. Parker,[1] Coby J Clarke,[2] Robert G. Palgrave,[3] Roger A Bennett,[1] Ricardo Grau-Crespo,[1] Kevin R. J. Lovelock[1]*

[1] Department of Chemistry, University of Reading, Reading, UK, RG6 6DX
[2] School of Chemistry, University of Nottingham, Nottingham, UK, NG7 2RD
[3] Department of Chemistry, University College London, UK, WC1H 0AJ
* k.r.j.lovelock@reading.ac.uk

1. Ionic Liquids Studied and Synthesis S2-S9
2. Data analysis. Peak fitting core level XP spectra and charge referencing S10-S11
3. Results. XPS: demonstrating purity S12-S18
4. Results. Anion-cation interaction strength scale for 39 different anions S19-S27
5. Results. Experimental versus calculated core XPS S28-S29
6. Results. Linear correlations of E_B and ESP S30
7. Results. Proving that size does not matter strongly to anion interaction strength S31
8. Results. $E_B(O_{anion}$ 1s) versus $E_B(N_{cation}$ 1s) S32
9. Results. $[A]^-$-dependent $[A]^-$-$[C]^+$ interactions S33-S35
10. Results. Anion-cation and anion-neutral molecule interactions S36-S38
11. References S39

1. Ionic Liquids Studied and Synthesis

Table S1 gives the ionic liquids (ILs) studied. All 43 ILs were liquid at room temperature, making XPS experiments relatively straightforward, as no heating was required for any IL studied here. $[C_8C_1Im]_2[Bi_2Cl_8]$, $[C_8C_1Im][SnBr_3]$, $[C_8C_1Im][InBr_4]$ and $[C_8C_1Im]_2[Zn_3Cl_8]$ were prepared using the following method. 1-Octyl-3-methylimidazolium chloride, $[C_8C_1Im]Cl$, and 1-octyl-3-methylimidazolium bromide, $[C_8C_1Im]Br$, were purchased from Iolitec, and dried on a Schlenk line at <2 × 10^{-2} mbar at 70 °C for 48 h before being transferred to a LABstar (MBraun) glovebox with <0.5 ppm of O_2 and H_2O. These viscous ILs were gently heated to 40 °C; next, 2-3 g of the appropriate IL was decanted into a series of 30 mL screw top glass vials. The desired amount of metal salt (ESI Table S2 for metal salt sources, used as received) was calculated and weighed into each vial, stirrer bars were added, and the temperature was raised to 70 °C. The vials were periodically handled to wash any solid particles from the sides until clear solutions persisted (typically < 48 h). For laboratory XPS measurements, the samples were mounted in air; exposure to air was limited to <10 minutes. $[C_8C_1Im][CF_3CO_2]$ and $[C_8C_1Im][FSI]$ were both purchased from Iolitec (custom-synthesis) and used as received.

Table S1. ILs investigated in this work.

IL no.	Abbreviation	Structure	Name
1	[C_8C_1Im]Cl	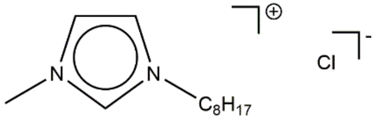	1–octyl–3–methylimidazolium chloride
2	[C_8C_1Im][CH_3CO_2]	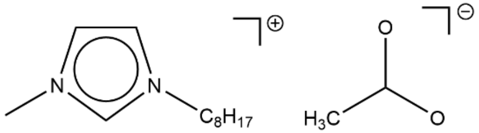	1–octyl–3–methylimidazolium acetate
3	[C_4C_1Im][$OcSO_4$]	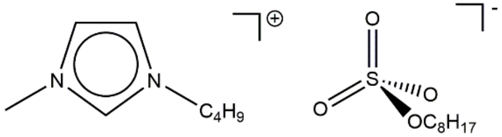	1–butyl–3–methylimidazolium octylsulfate
4	[C_8C_1Im]Br	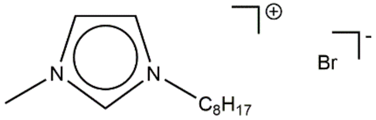	1–octyl–3–methylimidazolium bromide
5	[C_8C_1Im][HSO_4]	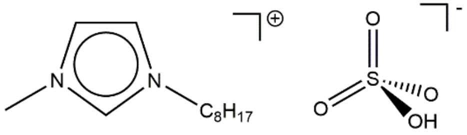	1–octyl–3–methylimidazolium hydrogensulfate
6	[C_8C_1Im]$_2$[$NiCl_4$]	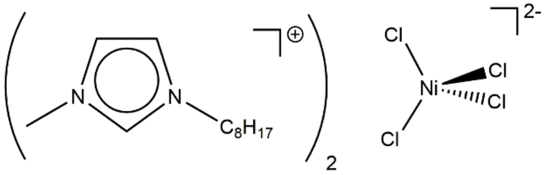	bis(1-octyl-3-methylimidazolium) tetrachloronickelate
7	[C_8C_1Im]$_2$[$CoCl_4$]	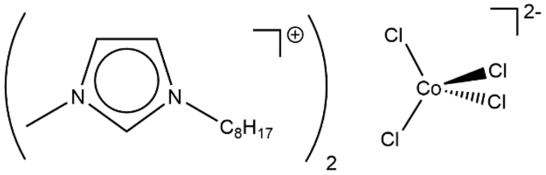	bis(1-octyl-3-methylimidazolium) tetrachlorocobaltate

8	$[C_8C_1Im]_2[FeCl_4]$	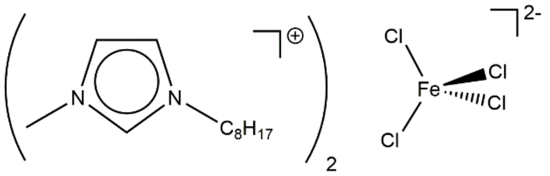	bis(1-octyl-3-methylimidazolium) tetrachloroferrate
9	$[C_8C_1Im]_2[ZnCl_4]$	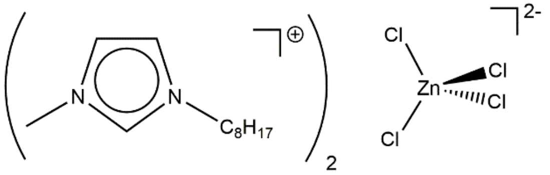	bis(1-octyl-3-methylimidazolium) tetrachlorozincate
10	$[C_8C_1Im][NO_3]$	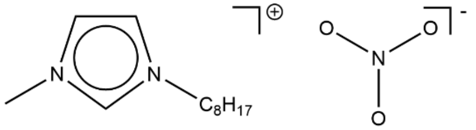	1–octyl–3–methylimidazolium nitrate
11	$[C_8C_1Im]_2[ZnCl_2Br_2]$	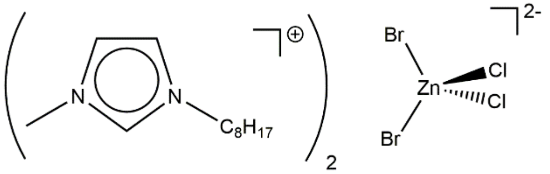	bis(1-octyl-3-methylimidazolium) dichlorodibromozincate
12	$[C_6C_1Im]I$	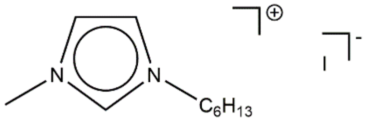	1–hexyl–3–methylimidazolium iodide
13	$[C_8C_1Im]_2[ZnBr_4]$	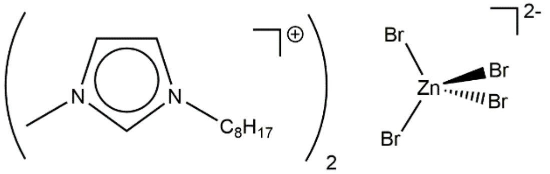	bis(1-octyl-3-methylimidazolium) tetrabromozincate
14	$[C_8C_1Im][SCN]$	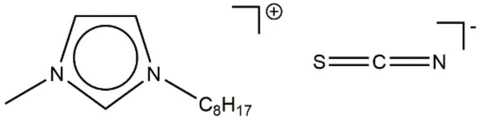	1–octyl–3–methylimidazolium thiocyanate
15	$[C_8C_1Im]_2[CoBr_4]$		bis(1-octyl-3-methylimidazolium) tetrabromocobaltate

16	$[C_8C_1Im][TfO]$	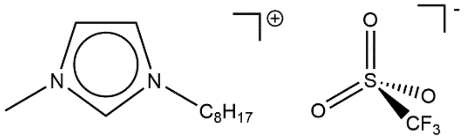	1–octyl–3–methylimidazolium trifluoromethylsulfonate
17	$[C_8C_1Im]_2[Bi_2Cl_8]$	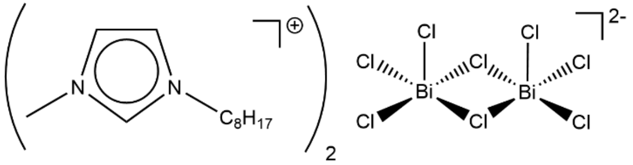	bis(1-octyl-3-methylimidazolium) octachlorodibismuthate
18	$[C_8C_1Im][CF_3CO_2]$	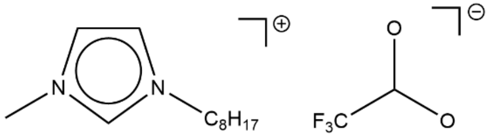	1–octyl–3–methylimidazolium trifluoroacetate
19	$[C_8C_1Im]_2[Zn_2Br_6]$	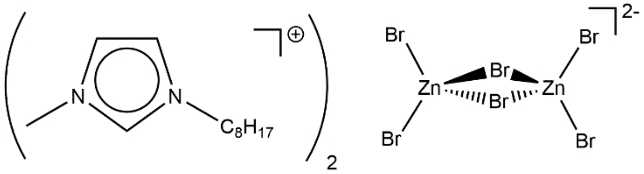	bis(1-octyl-3-methylimidazolium) hexabromodizincate
20	$[C_8C_1Im]_2[Zn_2Cl_6]$	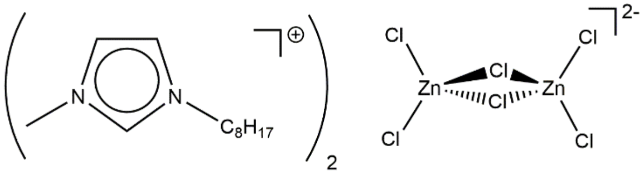	bis(1-octyl-3-methylimidazolium) hexachlorodizincate
21	$[C_8C_1Im][BF_4]$	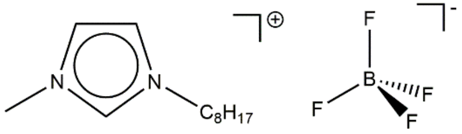	1–octyl–3–methylimidazolium tetrafluoroborate
22	$[C_8C_1Im][SnBr_3]$	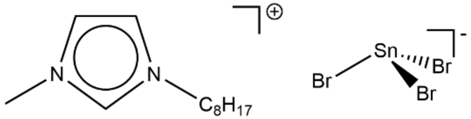	1-octyl-3-methylimidazolium tribromostannate

23	$[C_8C_1Im][SnCl_3]$	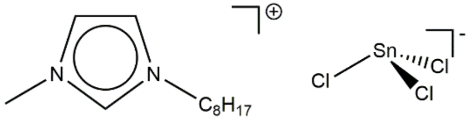	1-octyl-3-methylimidazolium trichlorostannate
24	$[C_4C_1Im][N(CN)_2]$	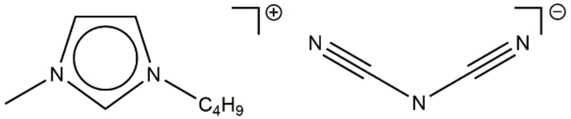	1–butyl–3–methylimidazolium dicyanamide
25	$[C_8C_1Im]_2[Zn_3Br_8]$	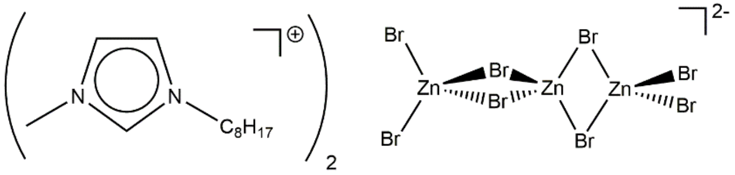	bis(1-octyl-3-methylimidazolium) octabromotrizincate
26	$[C_8C_1Im]_2[Zn_3Cl_8]$	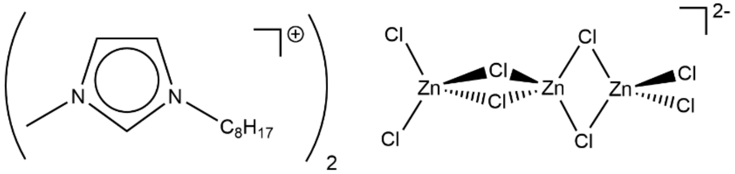	bis(1-octyl-3-methylimidazolium) octachlorootrizincate
27	$[C_8C_1Im]_2[Zn_4Br_{10}]$	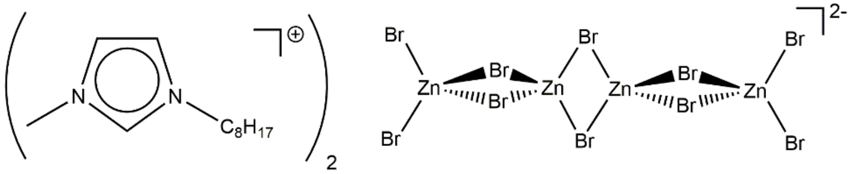	bis(1-octyl-3-methylimidazolium) decabromotetrazincate
28	$[C_8C_1Im]_2[Zn_4Cl_{10}]$	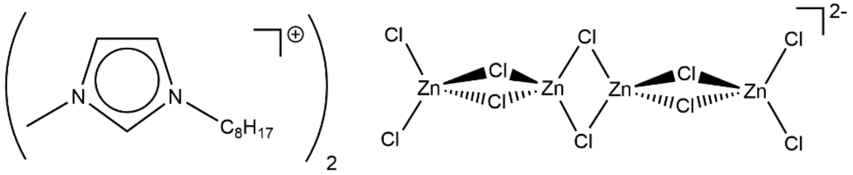	bis(1-octyl-3-methylimidazolium) decachlorotetrazincate [b]

29	$[C_8C_1Im][C(CN)_3]$	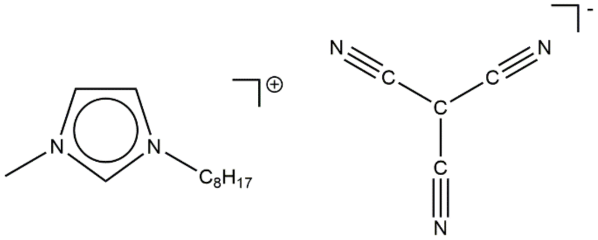	1–octyl–3–methylimidazolium tricyanomethanide
30	$[C_6C_1Im][B(CN)_4]$	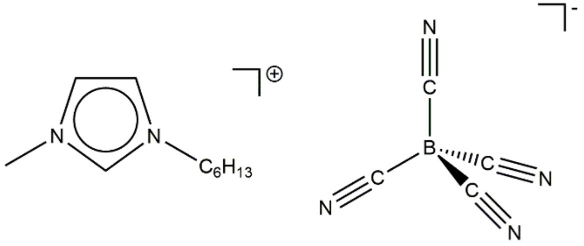	1–hexyl–3–methylimidazolium tetracyanoborate
31	$[C_8C_1Im][NTf_2]$	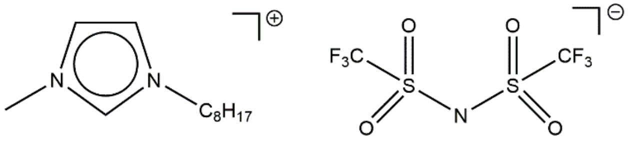	1–octyl–3–methylimidazolium bis[(trifluoromethane)sulfonyl]imide
32	$[C_4C_1Im][PF_6]$	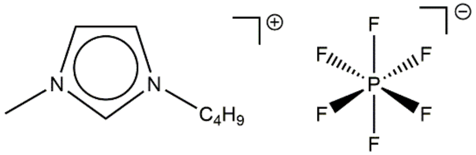	1-butyl-3-methylimidazolium hexafluorophosphate
33	$[C_8C_1Im][I_3]$	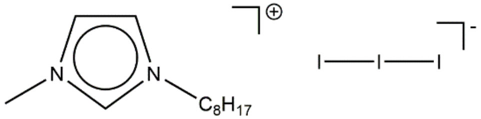	1-octyl-3-methylimidazolium triiodide
34	$[C_8C_1Im][FSI]$	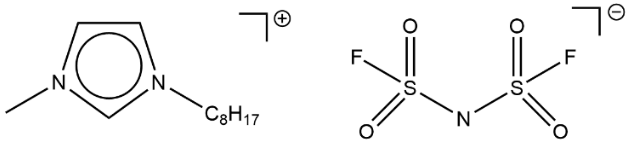	1-octyl-3-methylimidazolium bis(fluorosulfonyl)imide

35	$[C_8C_1Im][NPf_2]$	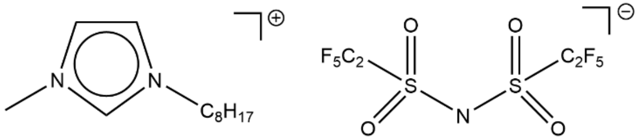	1-octyl-3-methylimidazolium bis(pentafluoroethylsulfonyl)imide
36	$[C_8C_1Im][SbF_6]$	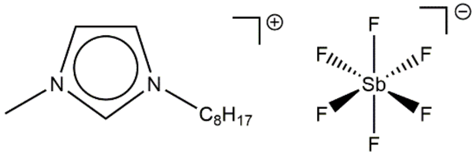	1-octyl-3-methylimidazolium hexafluoroantimonate
37	$[C_8C_1Im][InCl_4]$	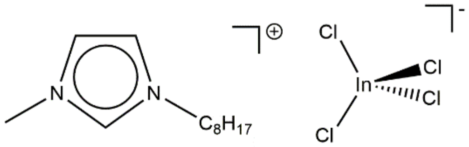	1-octyl-3-methylimidazolium tetrachloroindate
38	$[C_8C_1Im][InBr_4]$	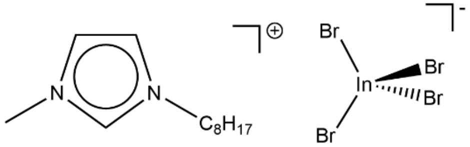	1-octyl-3-methylimidazolium tetrabromoindate
39	$[C_2C_1Im][FAP]$	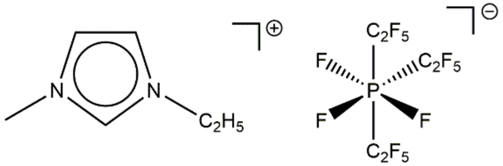	1-ethyl-3-methylimidazolium tris(pentafluoroethyl)trifluorophosphate [a]
40	$[N_{4,1,1,1}][NTf_2]$	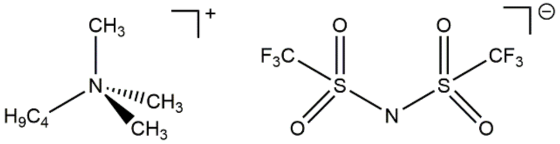	butyl(trimethyl)ammonium bis[(trifluoromethane)sulfonyl]imide
41	$[C_4Py][NTf_2]$	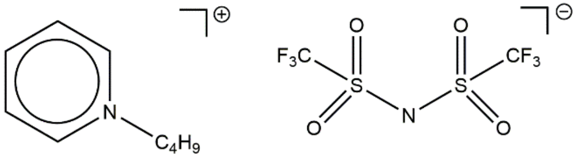	1-butylpyridinium bis[(trifluoromethane)sulfonyl]imide

42	$[P_{6,6,6,14}]Cl$	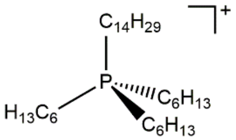	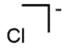	tetradecyl(trihexyl)phosphonium chloride
43	$[P_{6,6,6,14}][NTf_2]$	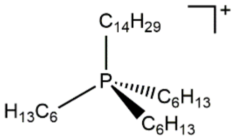	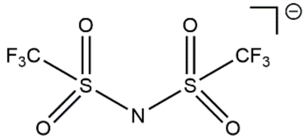	tetradecyl(trihexyl)phosphonium bis[(trifluoromethane)sulfonyl]imide

[a] $[FAP]^-$ drawn as meridional isomer, although facial isomer also expected to be present in the IL[1]
[b] $[Zn_4Cl_{10}]^{2-}$ and $[Zn_4Br_{10}]^{2-}$ drawn as linear, but supertetrahedron ring will also be present[2]

Table S2. Metal salts used to make halometallate ILs

Salt	Formula	Purity	Purchased from
bismuth(III) chloride	$BiCl_3$	anhydrous, powder, 99.998%	Sigma-Aldrich
tin(II) bromide	$SnBr_2$	99.2%	Alfa Aesar
indium(III) bromide	$InBr_3$	anhydrous, 99.99%,	Alfa Aesar
zinc(II) chloride	$ZnCl_2$	99.999% metals basis	Sigma-Aldrich

2. Data analysis. Peak fitting core level XP spectra and charge referencing

Peak fitting core level XP spectra is important for demonstrating purity (ESI Section 3). For this work peak fitting core level XP spectra is equally important for charge referencing. In ESI Table S3, the core orbital used for charge referencing is given, along with any constraints used. The constraints used were all explained in the ESI of reference [3].

Table S3. Experimental X-ray photoelectron spectroscopy (XPS) details on the charge correction applied for each ionic liquid, and any fitting constraints needed to fit the core orbital used for charge referencing

IL no.	Abbreviation	Charge referencing method used from reference [4]	Core orbital used for charge referencing	Fitting constraints used	E_B for core orbital used for charge referencing / eV	Rationale for choosing core orbital used for charge referencing
1	$[C_8C_1Im]Cl$	i	C_{alkyl} 1s	1:4:7 for C^2:C_{hetero}:C_{alkyl}	285.0	Long alkyl chain
2	$[C_8C_1Im][CH_3CO_2]$	i	C_{alkyl} 1s	1:4:7 for C^2:C_{hetero}:C_{alkyl}	285.0	Long alkyl chain
3	$[C_4C_1Im][OcSO_4]$	i	C_{alkyl} 1s	1:5:10 for C^2:C_{hetero}:C_{alkyl}	285.0	Long alkyl chain
4	$[C_8C_1Im]Br$	i	C_{alkyl} 1s	1:4:7 for C^2:C_{hetero}:C_{alkyl}	285.0	Long alkyl chain
5	$[C_8C_1Im][HSO_4]$	i	C_{alkyl} 1s	1:4:7 for C^2:C_{hetero}:C_{alkyl}	285.0	Long alkyl chain
6	$[C_8C_1Im]_2[NiCl_4]$	i	C_{alkyl} 1s	1:4:7 for C^2:C_{hetero}:C_{alkyl}	285.0	Long alkyl chain
7	$[C_8C_1Im]_2[CoCl_4]$	i	C_{alkyl} 1s	1:4:7 for C^2:C_{hetero}:C_{alkyl}	285.0	Long alkyl chain
8	$[C_8C_1Im]_2[FeCl_4]$	i	C_{alkyl} 1s	1:4:7 for C^2:C_{hetero}:C_{alkyl}	285.0	Long alkyl chain
9	$[C_8C_1Im]_2[ZnCl_4]$	i	C_{alkyl} 1s	1:4:7 for C^2:C_{hetero}:C_{alkyl}	285.0	Long alkyl chain
10	$[C_8C_1Im][NO_3]$	i	C_{alkyl} 1s	5:7 for C_{hetero}:C_{alkyl} [5]	285.0	Long alkyl chain
11	$[C_8C_1Im]_2[ZnCl_2Br_2]$	i	C_{alkyl} 1s	1:4:7 for C^2:C_{hetero}:C_{alkyl}	285.0	Long alkyl chain
12	$[C_6C_1Im]I$	ii	N_{cation} 1s	None	401.9	N_{cation} 1s for $[C_8C_1Im]I$ [6]
13	$[C_8C_1Im]_2[ZnBr_4]$	i	C_{alkyl} 1s	1:4:7 for C^2:C_{hetero}:C_{alkyl}	285.0	Long alkyl chain
14	$[C_8C_1Im][SCN]$	i	C_{alkyl} 1s	1:5:7 for C^2:C_{hetero}:C_{alkyl}	285.0	Long alkyl chain
15	$[C_8C_1Im]_2[CoBr_4]$	i	C_{alkyl} 1s	1:4:7 for C^2:C_{hetero}:C_{alkyl}	285.0	Long alkyl chain
16	$[C_8C_1Im][TfO]$	i	C_{alkyl} 1s	1:4:7 for C^2:C_{hetero}:C_{alkyl}	285.0	Long alkyl chain
17	$[C_8C_1Im]_2[Bi_2Cl_8]$	i	C_{alkyl} 1s	1:4:7 for C^2:C_{hetero}:C_{alkyl}	285.0	Long alkyl chain
18	$[C_8C_1Im][CF_3CO_2]$	i	C_{alkyl} 1s	1:4:7 for C^2:C_{hetero}:C_{alkyl}	285.0	Long alkyl chain
19	$[C_8C_1Im]_2[Zn_2Br_6]$	i	C_{alkyl} 1s	1:4:7 for C^2:C_{hetero}:C_{alkyl}	285.0	Long alkyl chain
20	$[C_8C_1Im]_2[Zn_2Cl_6]$	i	C_{alkyl} 1s	1:4:7 for C^2:C_{hetero}:C_{alkyl}	285.0	Long alkyl chain
21	$[C_8C_1Im][BF_4]$	i	C_{alkyl} 1s	1:4:7 for C^2:C_{hetero}:C_{alkyl}	285.0	Long alkyl chain
22	$[C_8C_1Im][SnBr_3]$	i	C_{alkyl} 1s	1:4:7 for C^2:C_{hetero}:C_{alkyl}	285.0	Long alkyl chain
23	$[C_8C_1Im][SnCl_3]$	i	C_{alkyl} 1s	1:4:7 for C^2:C_{hetero}:C_{alkyl}	285.0	Long alkyl chain
24	$[C_4C_1Im][N(CN)_2]$	ii	N_{cation} 1s	None	402.0	N_{cation} 1s for $[C_8C_1Im][N(CN)_2]$ [6]
25	$[C_8C_1Im]_2[Zn_3Br_8]$	i	C_{alkyl} 1s	1:4:7 for C^2:C_{hetero}:C_{alkyl}	285.0	Long alkyl chain
26	$[C_8C_1Im]_2[Zn_3Cl_8]$	i	C_{alkyl} 1s	1:4:7 for C^2:C_{hetero}:C_{alkyl}	285.0	Long alkyl chain
27	$[C_8C_1Im]_2[Zn_4Br_{10}]$	i	C_{alkyl} 1s	1:4:7 for C^2:C_{hetero}:C_{alkyl}	285.0	Long alkyl chain
28	$[C_8C_1Im]_2[Zn_4Cl_{10}]$	i	C_{alkyl} 1s	1:4:7 for C^2:C_{hetero}:C_{alkyl}	285.0	Long alkyl chain

29	$[C_8C_1Im][C(CN)_3]$	i	C_{alkyl} 1s	1:8:7 for C^2:C_{hetero}:C_{alkyl}	285.0	Long alkyl chain
30	$[C_6C_1Im][B(CN)_4]$	i	C_{alkyl} 1s	1:8:5 for C^2:C_{hetero}:C_{alkyl}	285.0	Long alkyl chain
31	$[C_8C_1Im][NTf_2]$	i	C_{alkyl} 1s	1:4:7 for C^2:C_{hetero}:C_{alkyl}	285.0	Long alkyl chain
32	$[C_4C_1Im][PF_6]$	ii	N_{cation} 1s	None	402.1	N_{cation} 1s for $[C_8C_1Im][PF_6]$
33	$[C_8C_1Im][I_3]$	i	C_{alkyl} 1s	1:4:7 for C^2:C_{hetero}:C_{alkyl}	285.0	Long alkyl chain
34	$[C_8C_1Im][FSI]$	i	C_{alkyl} 1s	1:4:7 for C^2:C_{hetero}:C_{alkyl}	285.0	Long alkyl chain
35	$[C_8C_1Im][NPf_2]$	i	C_{alkyl} 1s	5:7 for C_{hetero}:C_{alkyl} [5]	285.0	Long alkyl chain
36	$[C_8C_1Im][SbF_6]$	i	C_{alkyl} 1s	1:4:7 for C^2:C_{hetero}:C_{alkyl}	285.0	Long alkyl chain
37	$[C_8C_1Im][InCl_4]$	i	C_{alkyl} 1s	1:4:7 for C^2:C_{hetero}:C_{alkyl}	285.0	Long alkyl chain
38	$[C_8C_1Im][InBr_4]$	i	C_{alkyl} 1s	1:4:7 for C^2:C_{hetero}:C_{alkyl}	285.0	Long alkyl chain
39	$[C_2C_1Im][FAP]$	ii	CF_3 C 1s	None	293.14	CF_3 C 1s for $[C_8C_1Im][FAP]$ [5]
40	$[N_{4,1,1,1}][NTf_2]$	ii	F_{anion} 1s	None	688.8	F_{anion} 1s for $[N_{8,8,8,1}][NTf_2]$
41	$[C_4Py][NTf_2]$	ii	CF_3 C 1s	None	292.9	CF_3 C 1s for $[C_8Py][NTf_2]$
42	$[P_{6,6,6,14}]Cl$	i	C_{alkyl} 1s	4:28 for C_{hetero}:C_{alkyl}<br>FWHM(C_{hetero}) = FWHM(C_{alkyl})	285.0	Long alkyl chain
43	$[P_{6,6,6,14}][NTf_2]$	i	C_{alkyl} 1s	4:28 for C_{hetero}:C_{alkyl}<br>FWHM(C_{hetero}) = FWHM(C_{alkyl})	285.0	Long alkyl chain

3. Results. XPS: demonstrating purity

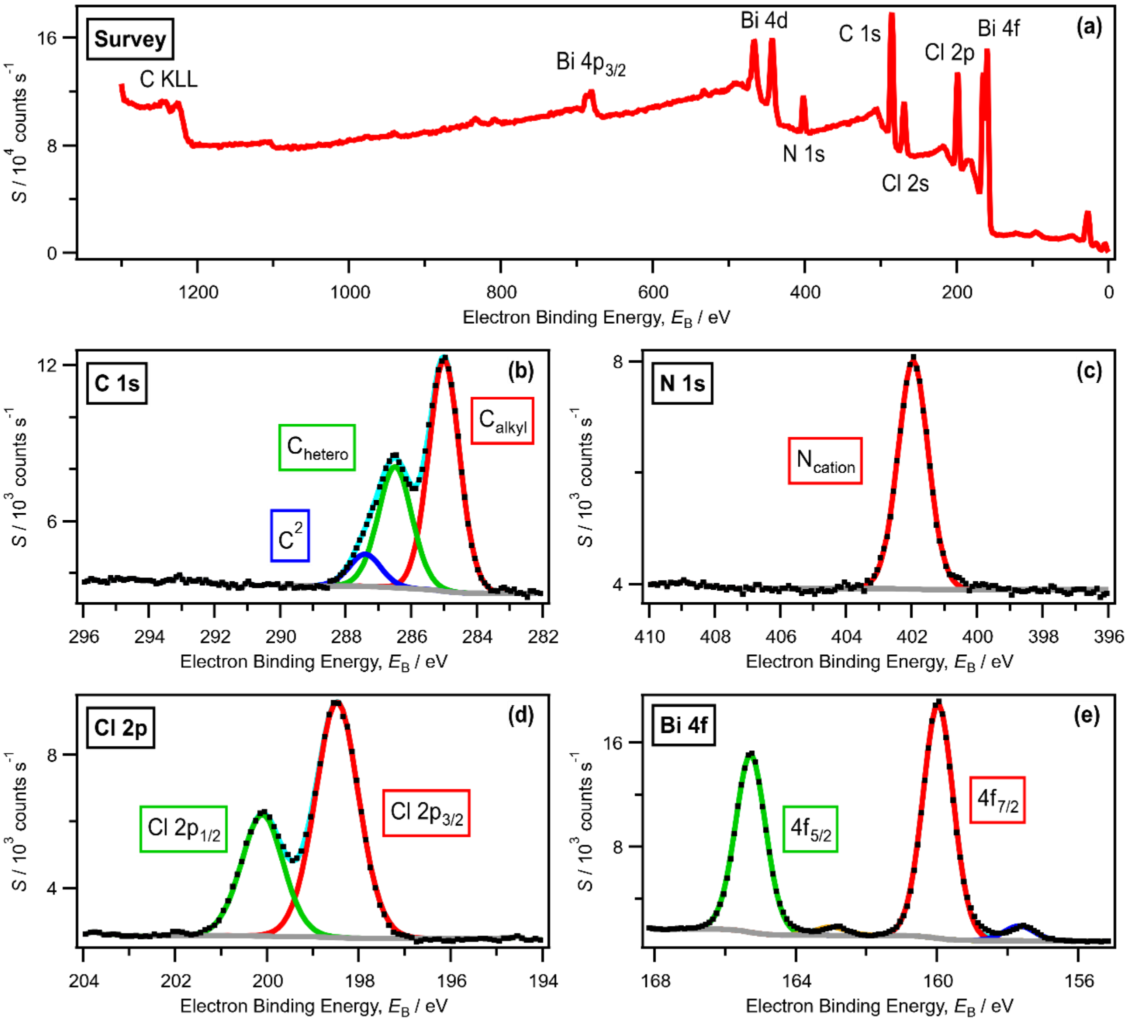

Figure S1. (a) Survey, (b-e) core XP spectra for $[C_8C_1Im]_2[Bi_2Cl_8]$ recorded on laboratory–based XPS apparatus at $h\nu = 1486.6$ eV. All XP spectra were charge referenced using the method outlined in ESI Section 2.

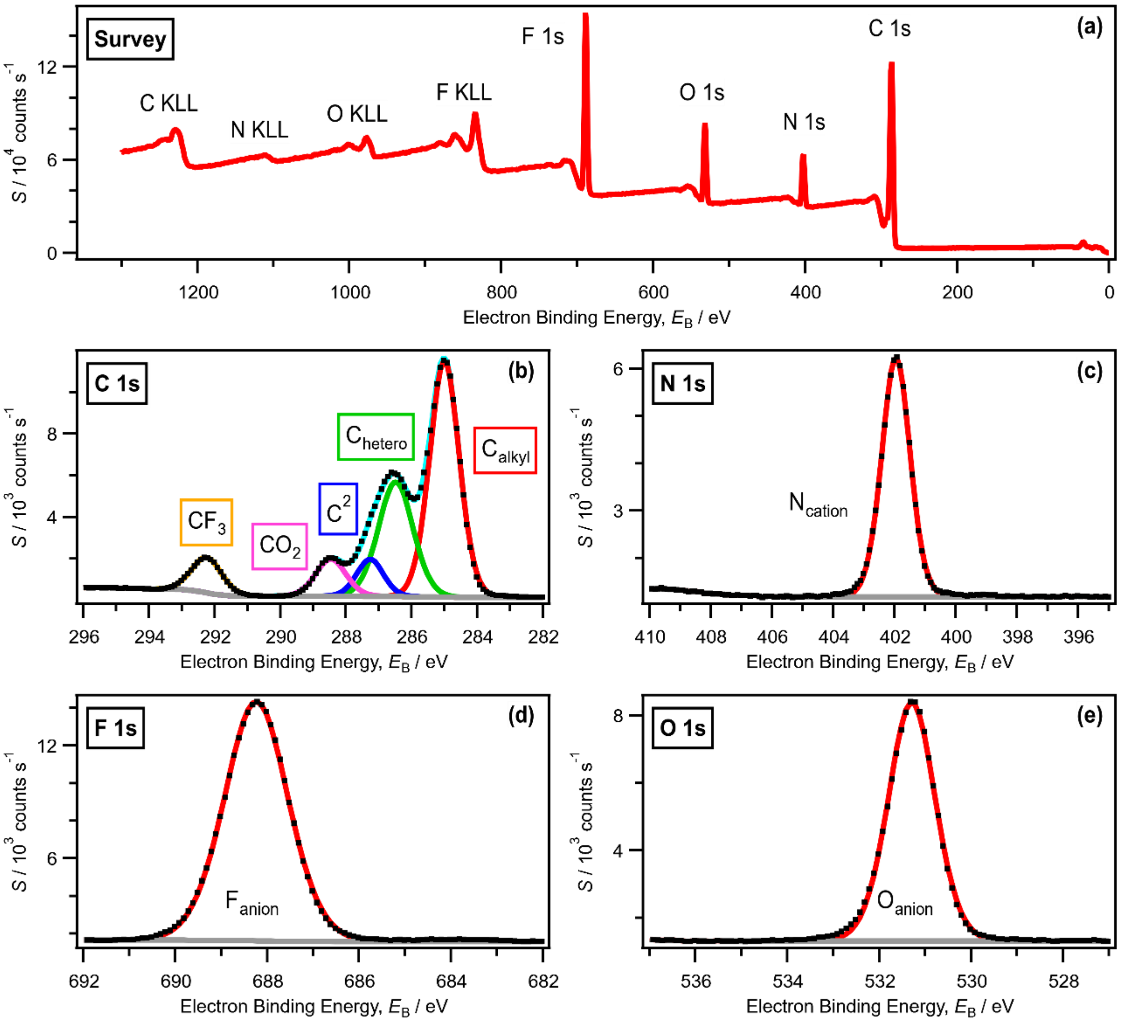

Figure S2. (a) Survey, (b-e) core XP spectra for [C_8C_1Im][CF_3CO_2] recorded on laboratory–based XPS apparatus at $h\nu$ = 1486.6 eV. All XP spectra were charge referenced using the method outlined in ESI Section 2.

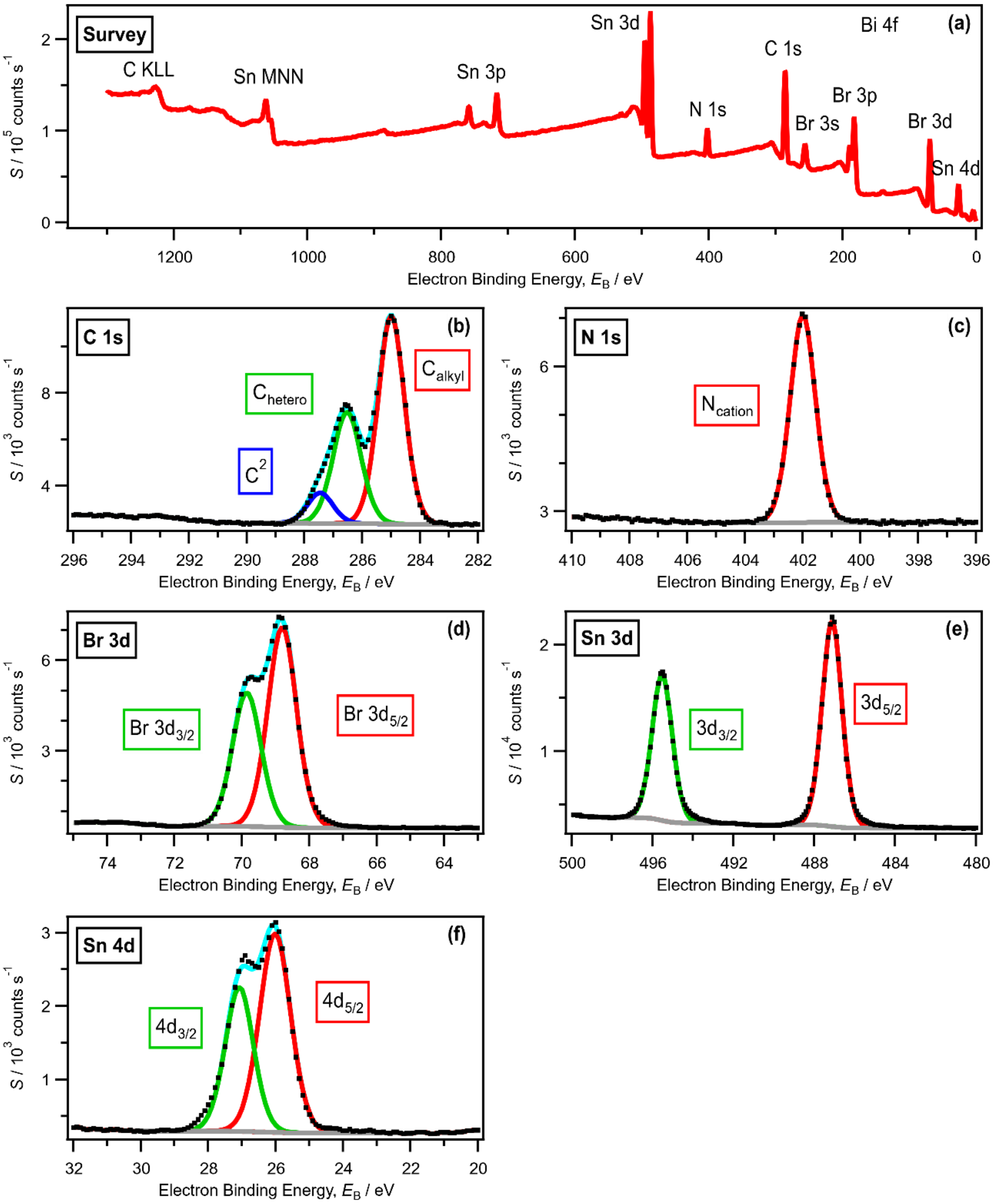

Figure S3. (a) Survey, (b-f) core XP spectra for [C_8C_1Im][$SnBr_3$] recorded on laboratory–based XPS apparatus at $h\nu$ = 1486.6 eV. All XP spectra were charge referenced using the method outlined in ESI Section 2.

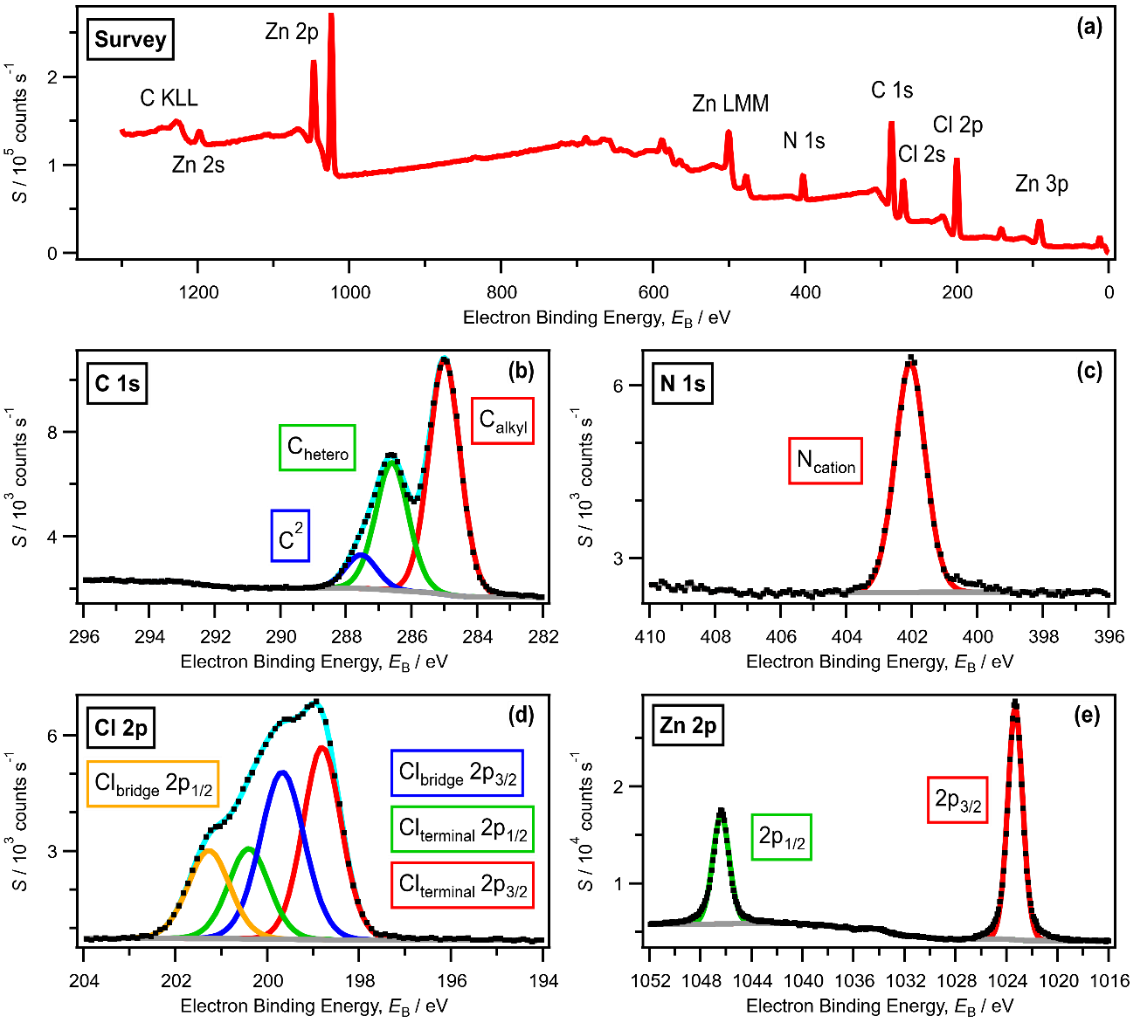

Figure S4. (a) Survey, (b-e) core XP spectra for $[C_8C_1Im]_2[Zn_3Cl_8]$ recorded on laboratory–based XPS apparatus at $h\nu$ = 1486.6 eV. All XP spectra were charge referenced using the method outlined in ESI Section 2.

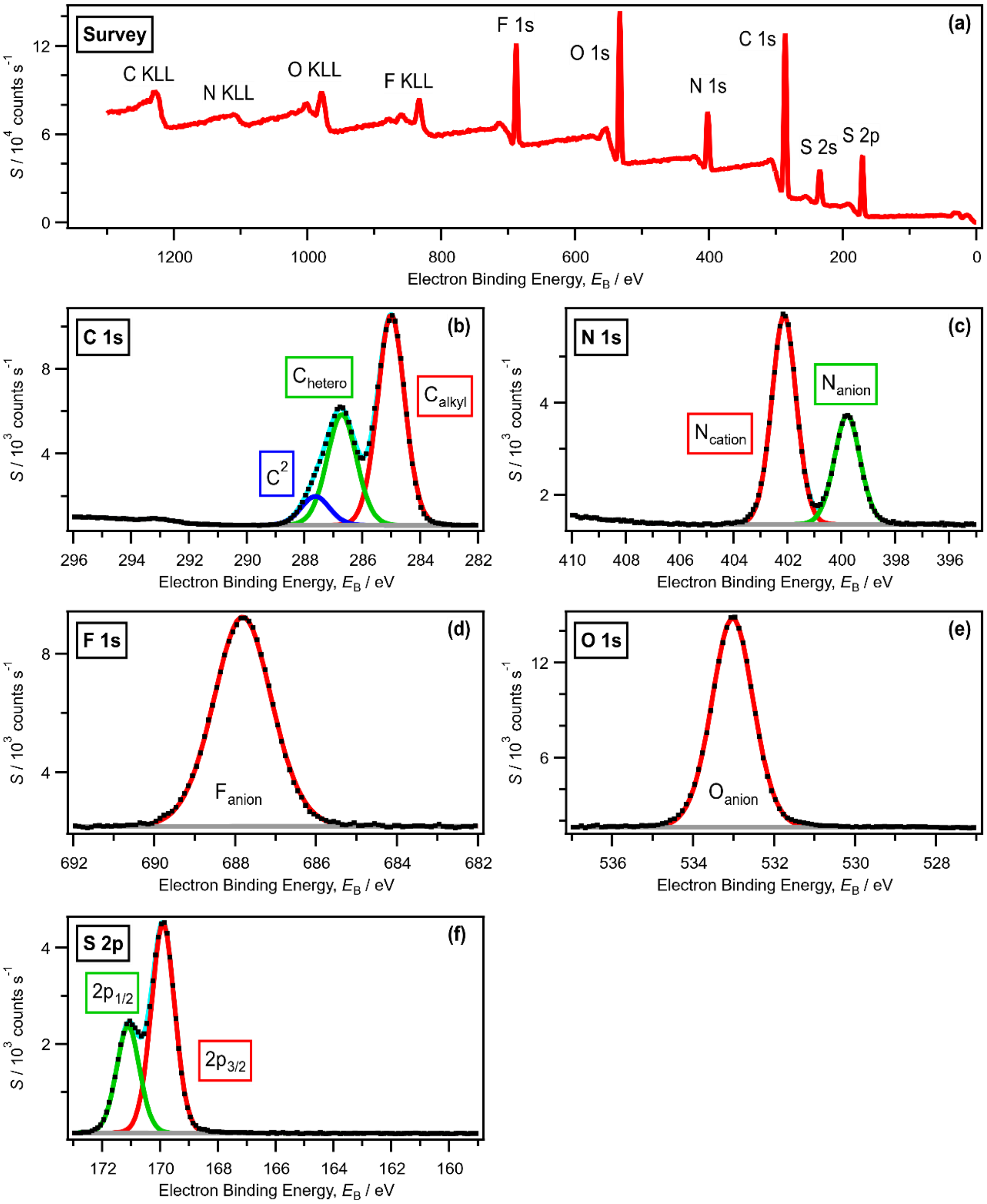

Figure S5. (a) Survey, (b-f) core XP spectra for $[C_8C_1Im][FSI]$ recorded on laboratory–based XPS apparatus at $h\nu$ = 1486.6 eV. All XP spectra were charge referenced using the method outlined in ESI Section 2.

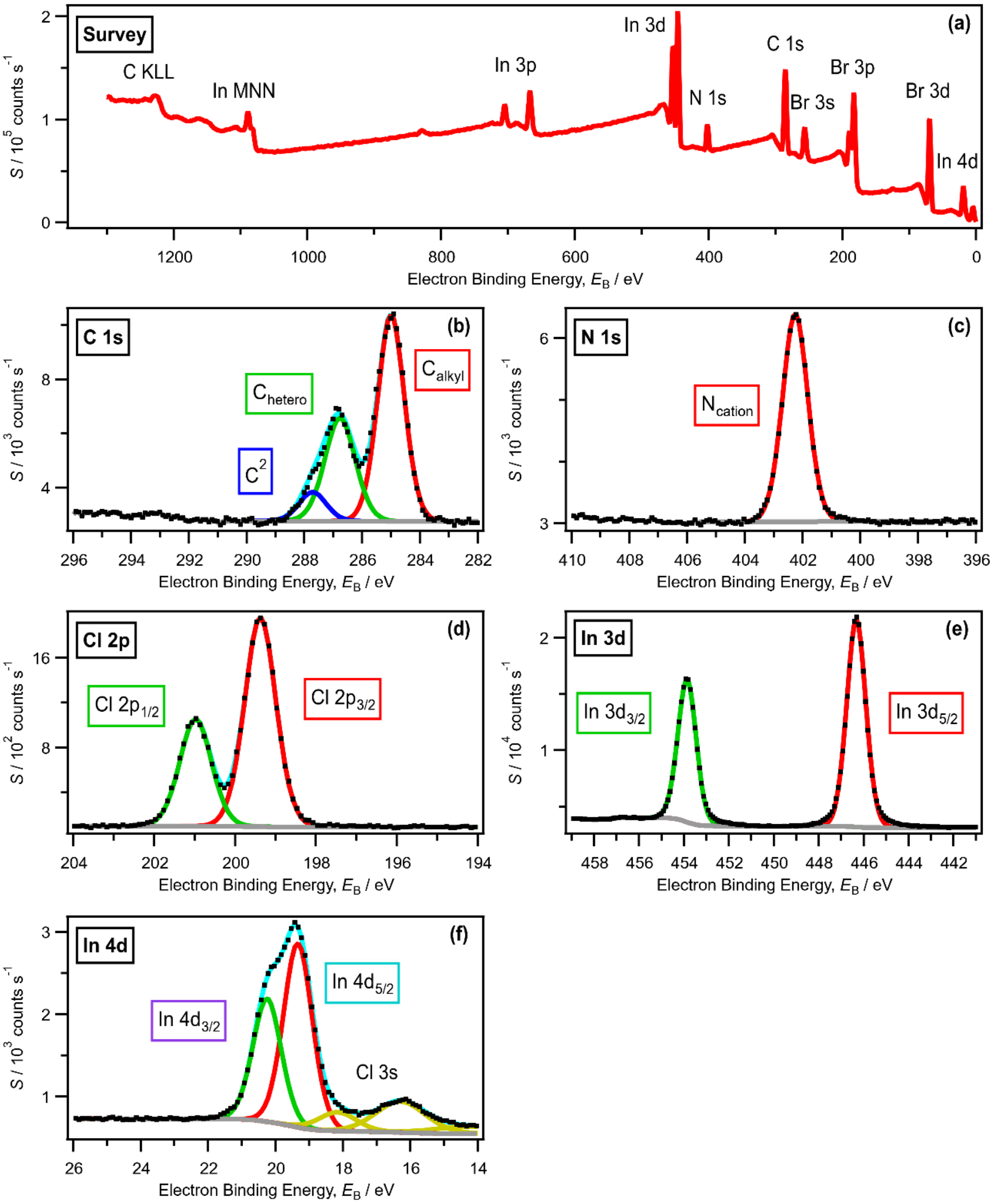

Figure S6. (a) Survey, (b-f) core XP spectra for $[C_8C_1Im][InBr_4]$ recorded on laboratory–based XPS apparatus at $h\nu$ = 1486.6 eV. All XP spectra were charge referenced using the method outlined in ESI Section 2.

Table S4. Measured experimental and nominal (in brackets) stoichiometries for the ionic liquids studied in this work, recorded at $h\nu$ = 1486.6 eV.

			C 1s	N 1s	Cl $2p_{3/2}$	Br $3d_{5/2}$	Zn $2p_{3/2}$	In $3d_{5/2}$	Sn $3d_{5/2}$	Bi $4f_{7/2}$
IL no.	Abbreviation	RSF [a]	0.205	0.350	0.367	0.330	4.800	3.900	4.300	4.250
	$[C_8C_1Im]_2[Bi_2Cl_8]$	Measured (nominal)	13.1 (12)	2.0 (2)	3.3 (4)					0.7 (1)
	$[C_8C_1Im][SnBr_3]$	Measured (nominal)	12.2 (12)	1.9 (2)		3.1 (3)			0.8 (1)	
	$[C_8C_1Im]_2[Zn_3Cl_8]$	Measured (nominal)	25.3 (24)	3.7 (4)	8.0 (8)		2.1 (3)			
	$[C_8C_1Im][InBr_4]$	Measured (nominal)	12.9 (12)	2.0 (2)		3.2 (4)		0.8 (1)		

[a] RSF = relative sensitivity factors, taken from reference [7] for C 1s, N 1s, Cl $2p_{3/2}$, Br $3d_{5/2}$ and from reference [8] for Zn $2p_{3/2}$, In $3d_{5/2}$, Sn $3d_{5/2}$ and Bi $4f_{7/2}$

The measured experimental and nominal stoichiometries (ESI Table S4) match well for all six ILs newly investigated here. Differences, especially the larger carbon values relative to other elements, are likely due to differences in relative sensitivity factors (RSF) values, as these RSF values were not tuned specially to the Reading XPS apparatus (in references [7, 9] RSF values were tuned specially to the XPS apparatus used in those studies). This match, along with the high quality XP spectra given in ESI Figure S1 to ESI Figure S6, demonstrates the high purity of the IL samples newly presented here.

4. Results. Anion-cation interaction strength scale for 39 different anions

Table S5. $E_B(N_{cation}$ 1s), $E_B(Cl_{terminal}$ $2p_{3/2})$, $E_B(Br_{terminal}$ $3d_{5/2})$, $E_B(O_{anion}$ 1s) for 39 ILs $[C_nC_1Im][A]$ and two other ILs studied here, including the reference where the values were first published, calculated anion molecular volume, V_{mol}, taken from reference [10] (plus calculated anion molar concentration and calculated $[C_8C_1Im]$[anion] molar concentration calculated from data in reference [10]) for 17 anions

IL	$E_B(N_{cation}$ 1s) / eV	$E_B(Cl_{terminal}$ $2p_{3/2})$ / eV	$E_B(Br_{terminal}$ $3d_{5/2})$ / eV	$E_B(O_{anion}$ 1s) / eV	Reference	V_{mol} / nm^3	calculated anion molar concentration / mol dm^{-3}	calculated $[C_8C_1Im]$[anion] molar concentration / mol dm^{-3}
$[C_8C_1Im]Cl$	401.68	196.96			[4]	0.0449	37.03	4.37
$[C_8C_1Im][CH_3CO_2]$	401.69			530.32	[11]	0.0897	18.51	3.91
$[C_4C_1Im][OcSO_4]$	401.71				[3]	0.3179	5.22	2.54
$[C_8C_1Im]Br$	401.71		67.41		[4]	0.0450	36.87	4.37
$[C_8C_1Im][HSO_4]$	401.78			531.63	[3]			
$[C_8C_1Im]_2[NiCl_4]$	401.81	198.16			[4]			
$[C_8C_1Im]_2[CoCl_4]$	401.81	198.18			[4]			
$[C_8C_1Im]_2[FeCl_4]$	401.82	198.20			[4]			
$[C_8C_1Im]_2[ZnCl_4]$	401.82	198.16			[4]			
$[C_8C_1Im][NO_3]$	401.83			532.10	[5]	0.0667	24.89	4.13
$[C_8C_1Im]_2[ZnCl_2Br_2]$	401.84				[4]			
$[C_6C_1Im]I$	401.86				[3]	0.0840	19.78	3.96
$[C_8C_1Im]_2[ZnBr_4]$	401.88		68.47		[4]			
$[C_8C_1Im][SCN]$	401.88				[3]	0.0831	19.98	3.97
$[C_8C_1Im]_2[CoBr_4]$	401.91		68.49		[4]			
$[C_8C_1Im][TfO]$	401.95			532.12	[3]	0.1453	11.43	3.46
$[C_8C_1Im]_2[Bi_2Cl_8]$	401.95				here			
$[C_8C_1Im][CF_3CO_2]$	401.96			531.29	here			
$[C_8C_1Im]_2[Zn_2Br_6]$	401.96		68.91		[4]			
$[C_8C_1Im]_2[Zn_2Cl_6]$	401.97	198.66			[4]			
$[C_8C_1Im][BF_4]$	401.98				[3]	0.0891	18.64	3.91
$[C_8C_1Im][SnBr_3]$	401.99		68.80		here			
$[C_8C_1Im][SnCl_3]$	402.00	198.58			[4]			
$[C_4C_1Im][N(CN)_2]$	402.00				[3]	0.0987	16.84	3.83
$[C_8C_1Im]_2[Zn_3Br_8]$	402.03		69.01		[4]			
$[C_8C_1Im]_2[Zn_3Cl_8]$	402.05				here			
$[C_8C_1Im]_2[Zn_4Br_{10}]$	402.06		69.03		[4]			
$[C_8C_1Im]_2[Zn_4Cl_{10}]$	402.06	198.87			[4]			
$[C_8C_1Im][C(CN)_3]$	402.07				[3]	0.1405	11.82	3.49
$[C_6C_1Im][B(CN)_4]$	402.07				[3]			

[C$_8$C$_1$Im][NTf$_2$]	402.10			532.66	3	0.2618	6.35	2.78
[C$_4$C1Im][PF$_6$]	402.10				4	0.1224	13.57	3.63
[C$_8$C$_1$Im][I$_3$]	402.11				12			
[C$_8$C$_1$Im][FSI]	402.13			533.03	here			
[C$_8$C$_1$Im][NPf$_2$]	402.14			532.78	5	0.3501	4.74	2.42
[C$_8$C$_1$Im][SbF$_6$]	402.15				13	0.1436	11.57	3.47
[C$_8$C$_1$Im][InCl$_4$]	402.19	199.38			4	0.1963	8.46	3.12
[C$_8$C$_1$Im][InBr$_4$]	402.26		69.57		here			
[C$_2$C$_1$Im][FAP]	402.27				4	0.3754	4.42	2.34
[N$_{4,1,1,1}$][NTf$_2$]	402.90				4			
[C$_4$Py][NTf$_2$]	402.54				4			

Table S6. E_B(N$_{anion}$ 1s) for eight ILs studied here, including the reference where the values were first published

IL	E_B(N$_{anion}$ 1s) / eV	Reference
[C$_8$C$_1$Im][SCN]	397.79	4
[C$_4$C$_1$Im][N(CN)$_2$]	398.36	4
[C$_8$C$_1$Im][C(CN)$_3$]	398.84	4
[C$_6$C$_1$Im][B(CN)$_4$]	399.75	4
[C$_8$C$_1$Im][NTf$_2$]	399.46	4
[C$_8$C$_1$Im][NPf$_2$]	399.50	5
[C$_8$C$_1$Im][FSI]	399.79	here
[C$_8$C$_1$Im][NO$_3$]	406.45	5

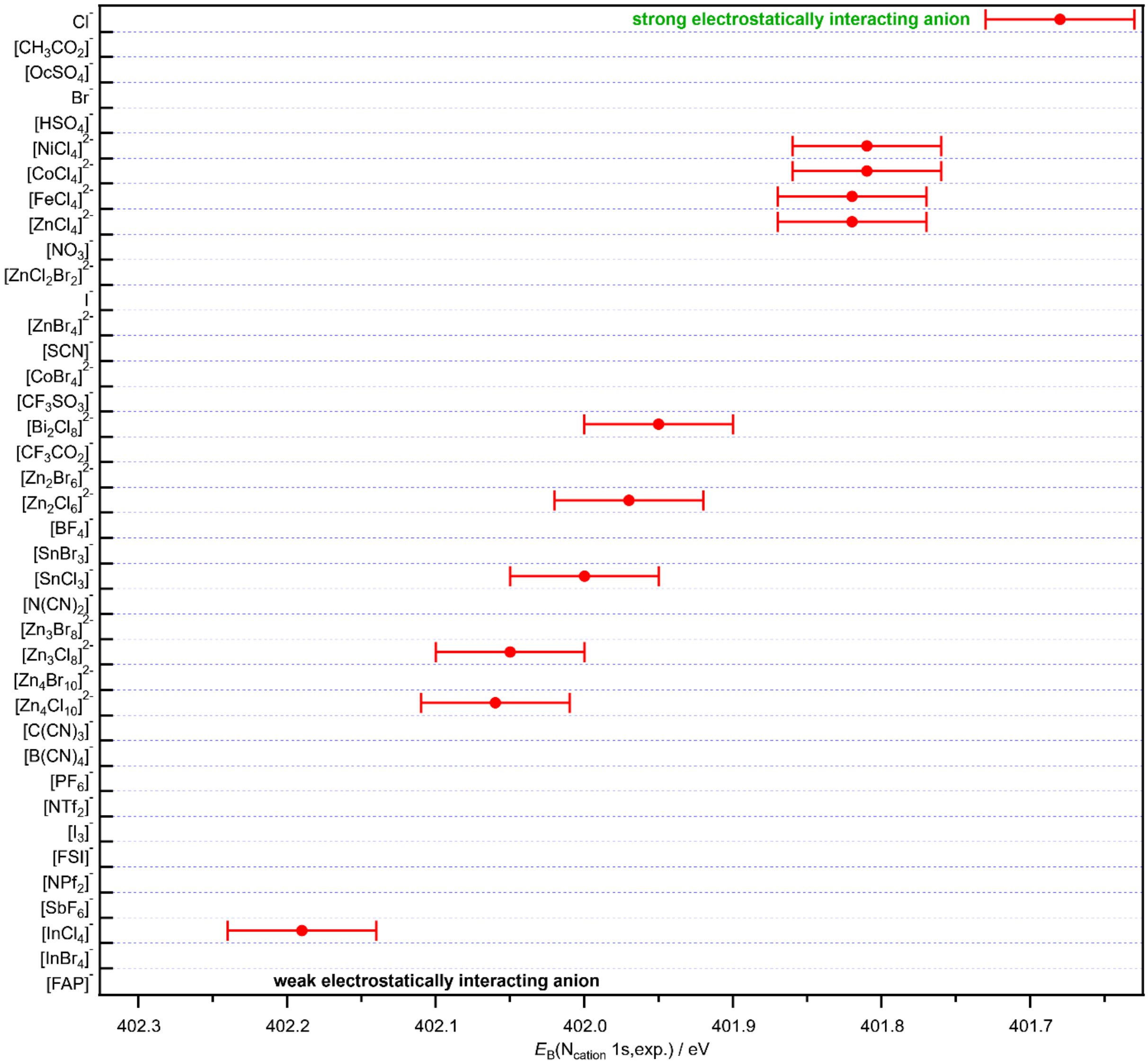

Figure S7. E_B(N_{cation} 1s) for 11 different Cl-containing anions (see ESI Table S5 for the numerical values). The estimated error is ±0.05 eV. Data from this paper apart from $[NO_3]^-$,[5] $[NPf_2]^-$,[5] $[CH_3CO_2]^-$,[14] $[SbF_6]^-$,[13] $[I_3]^-$.[12] Large E_B(N_{cation} 1s) = weak electrostatically interacting anion and small E_B(N_{cation} 1s) = strong electrostatically interacting anion.

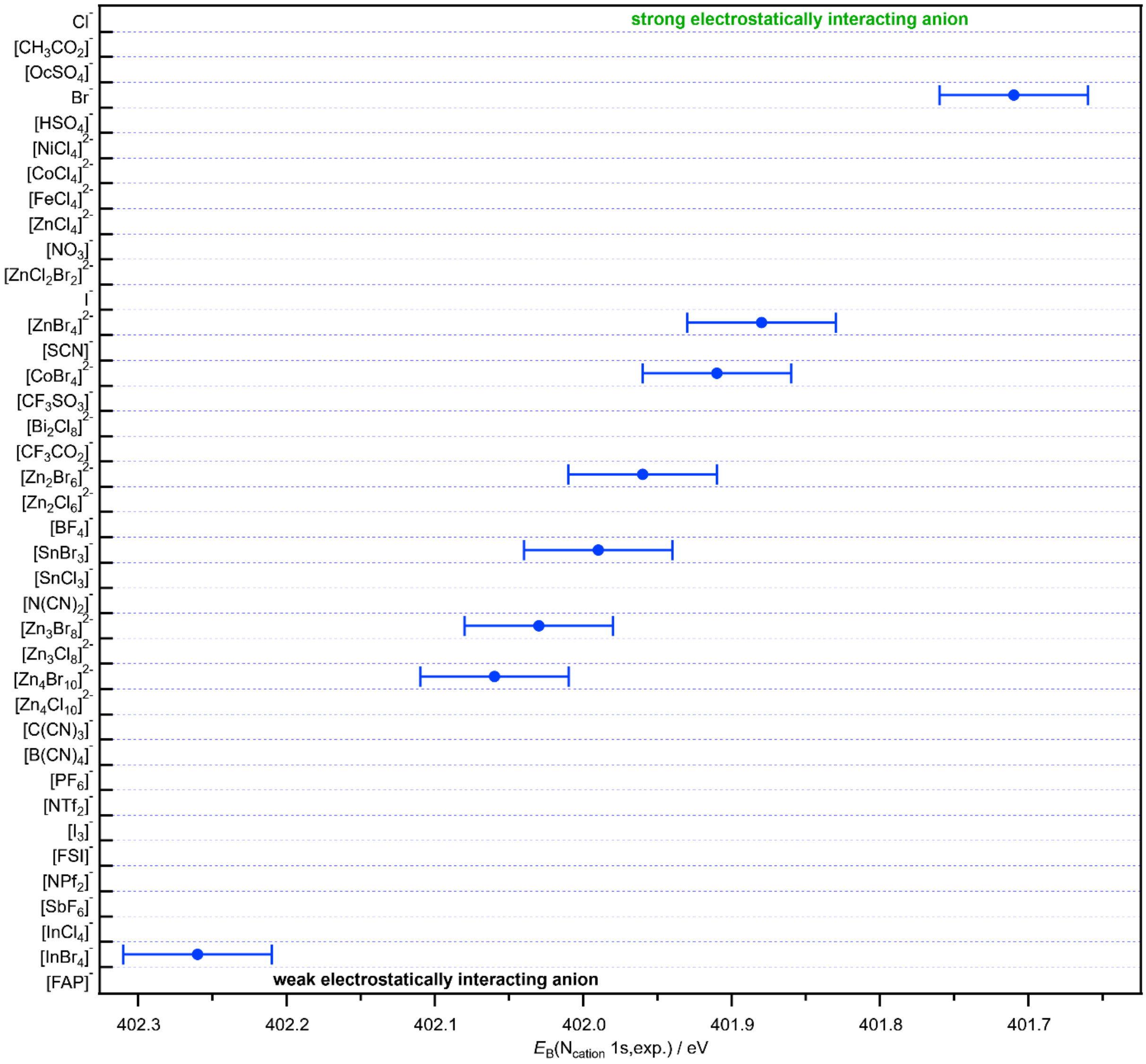

Figure S8. E_B(N_{cation} 1s) for eight different Br-containing anions (see ESI Table S5 for the numerical values). The estimated error is ±0.05 eV. Data from this paper apart from $[NO_3]^-$,[5] $[NPf_2]^-$,[5] $[CH_3CO_2]^-$,[14] $[SbF_6]^-$,[13] $[I_3]^-$.[12] Large E_B(N_{cation} 1s) = weak electrostatically interacting anion and small E_B(N_{cation} 1s) = strong electrostatically interacting anion.

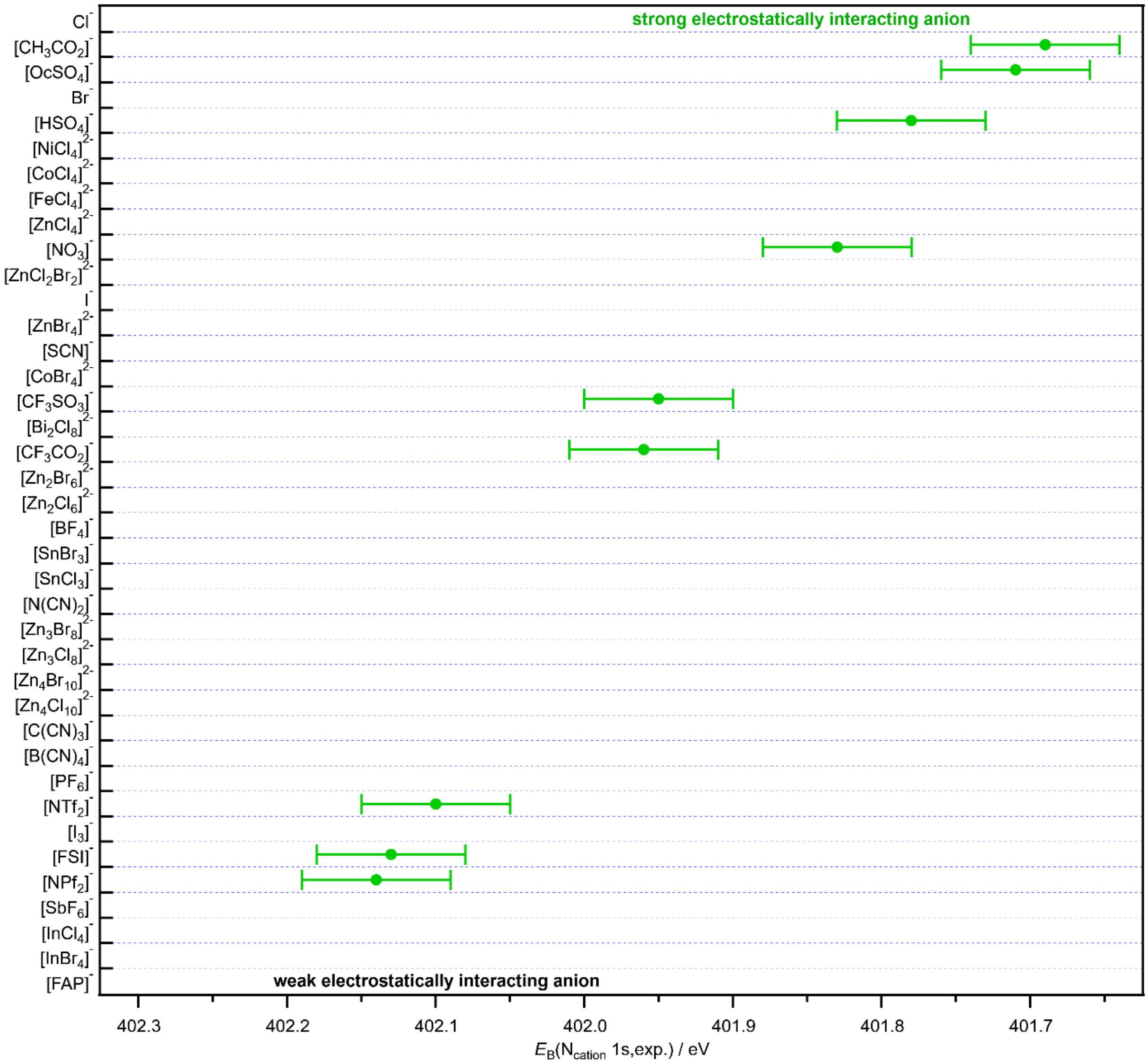

Figure S9. E_B(N_{cation} 1s) for nine different O-containing anions (see ESI Table S5 for the numerical values). The estimated error is ±0.05 eV. Data from this paper apart from $[NO_3]^-$,[5] $[NPf_2]^-$,[5] $[CH_3CO_2]^-$,[14] $[SbF_6]^-$,[13] $[I_3]^-$.[12] Large E_B(N_{cation} 1s) = weak electrostatically interacting anion and small E_B(N_{cation} 1s) = strong electrostatically interacting anion.

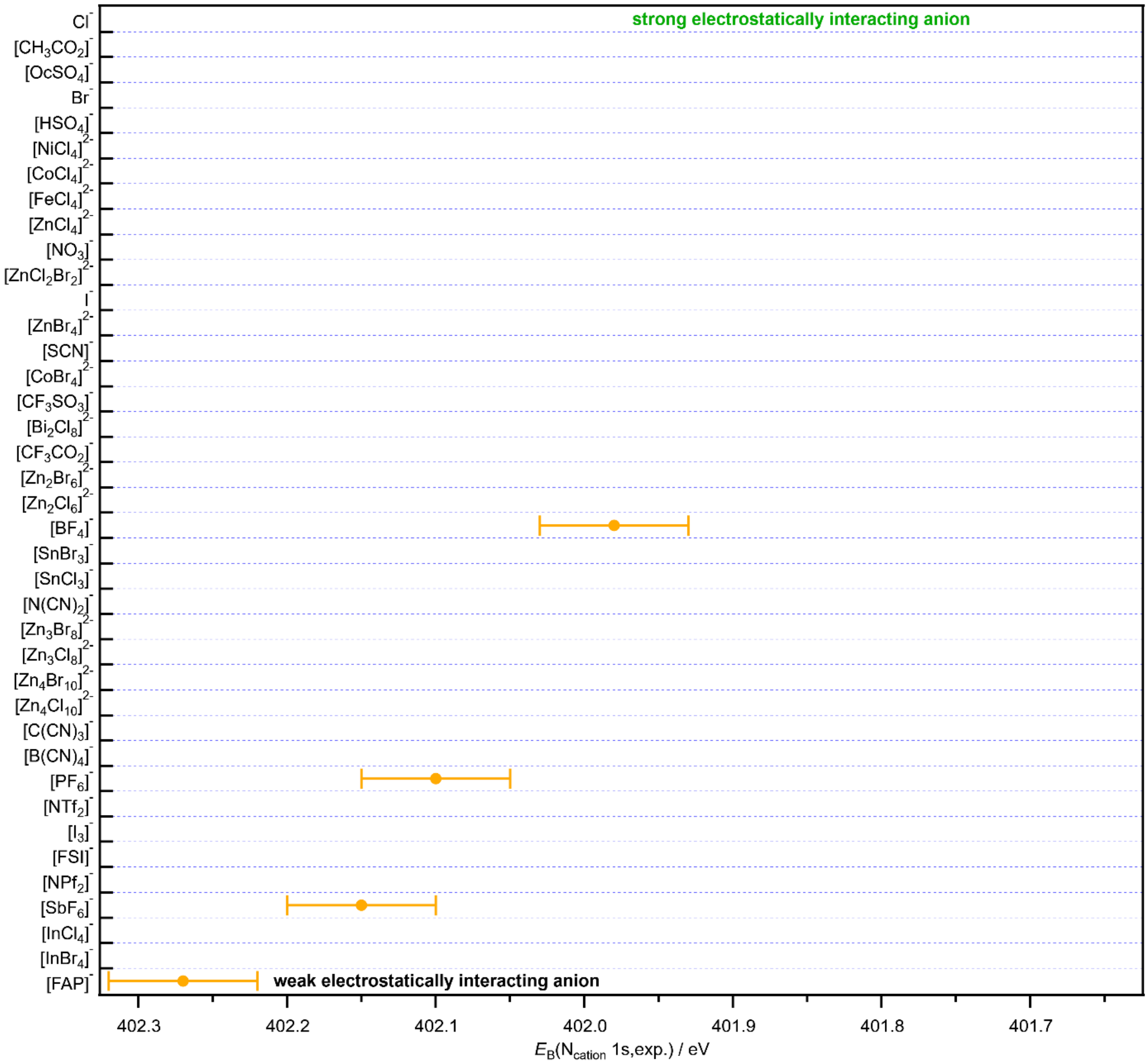

Figure S10. E_B(N_{cation} 1s) for four different F-containing anions (see ESI Table S5 for the numerical values). The estimated error is ±0.05 eV. Data from this paper apart from $[NO_3]^-$,[5] $[NPf_2]^-$,[5] $[CH_3CO_2]^-$,[14] $[SbF_6]^-$,[13] $[I_3]^-$.[12] Large E_B(N_{cation} 1s) = weak electrostatically interacting anion and small E_B(N_{cation} 1s) = strong electrostatically interacting anion.

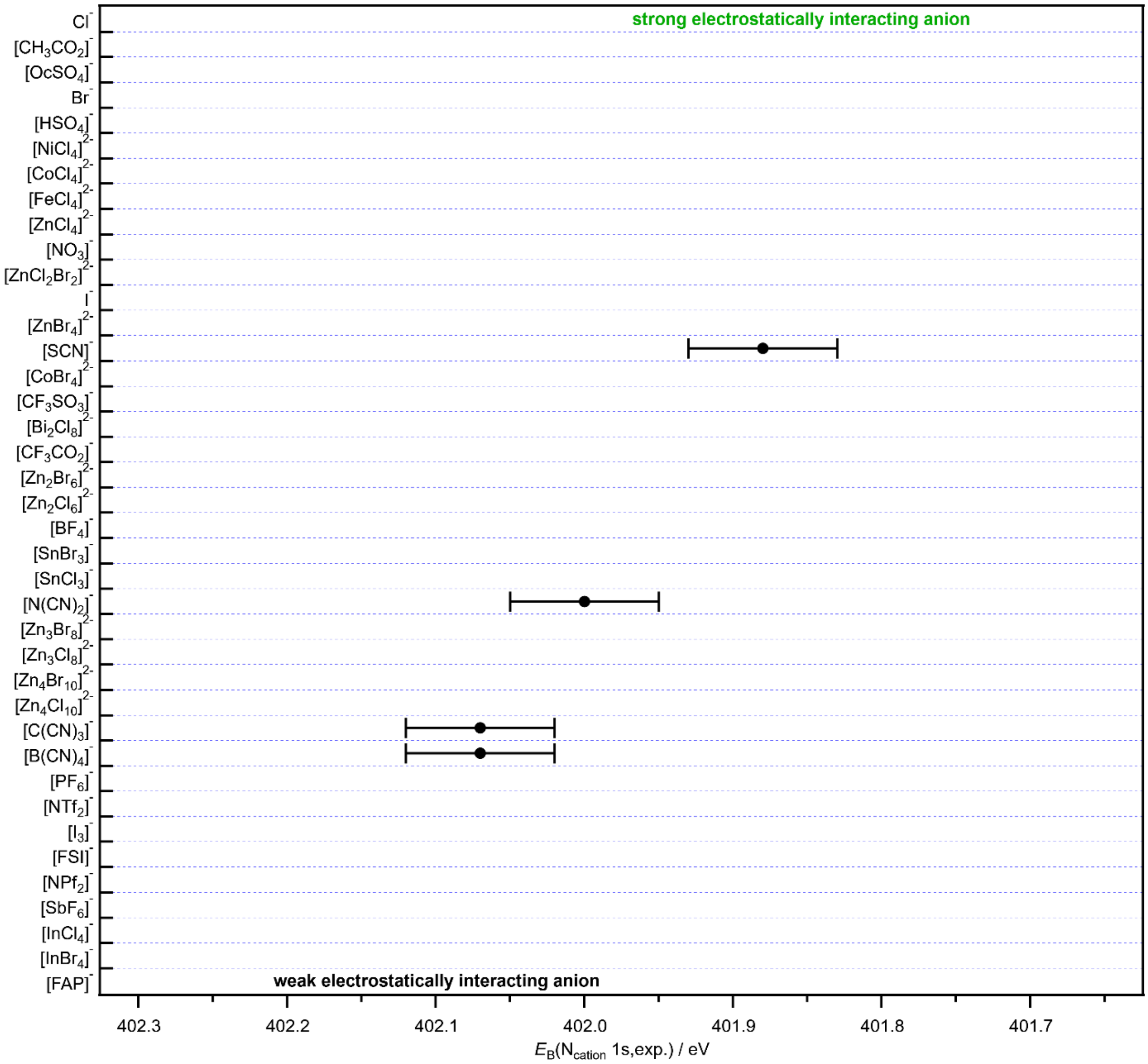

Figure S11. E_B(N_{cation} 1s) for four different N-containing anions (see ESI Table S5 for the numerical values). The estimated error is ±0.05 eV. Data from this paper apart from $[NO_3]^-$,[5] $[NPf_2]^-$,[5] $[CH_3CO_2]^-$,[14] $[SbF_6]^-$,[13] $[I_3]^-$.[12] Large E_B(N_{cation} 1s) = weak electrostatically interacting anion and small E_B(N_{cation} 1s) = strong electrostatically interacting anion.

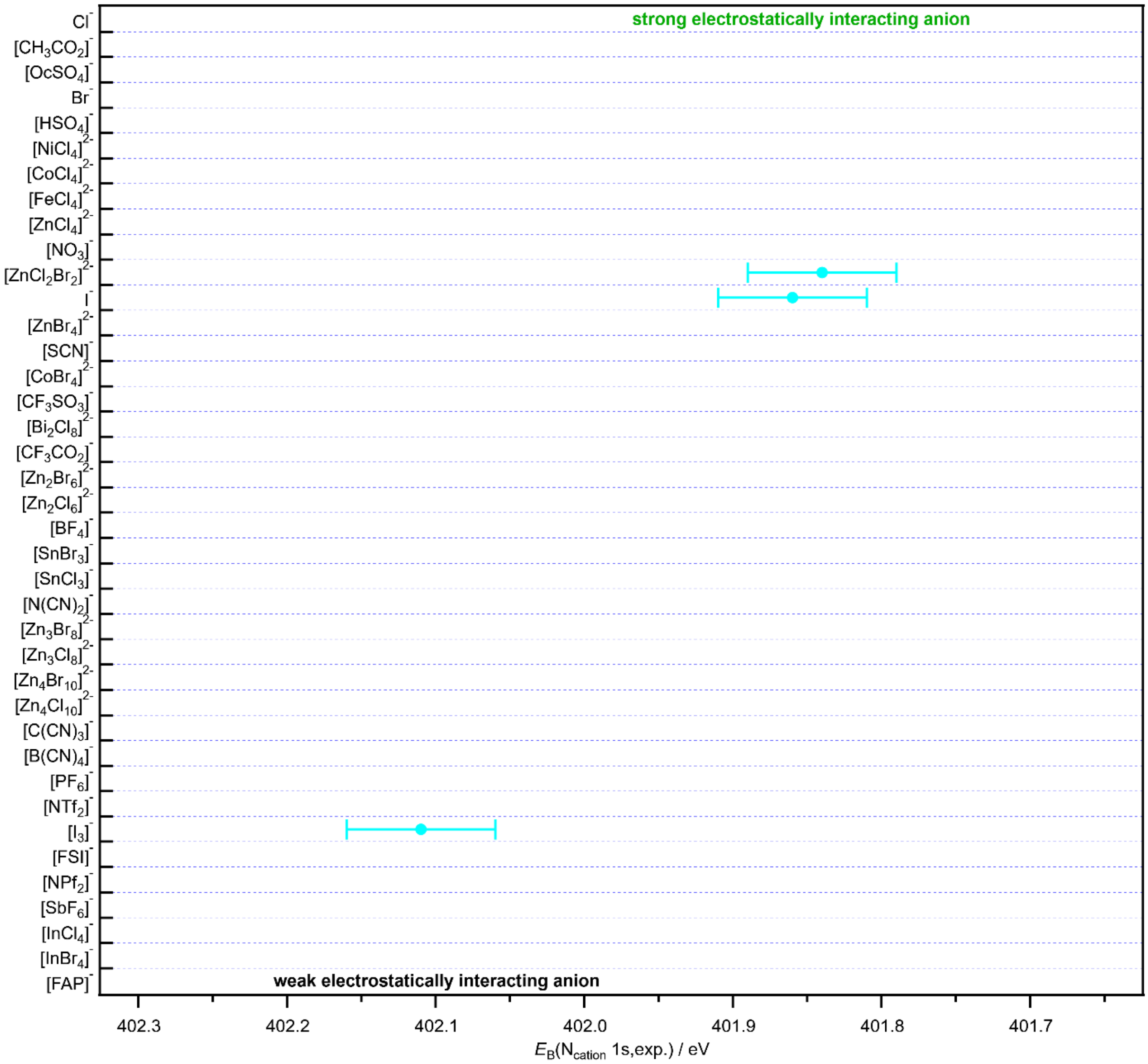

Figure S12. E_B(N_{cation} 1s) for three different halide-containing anions (see ESI Table S5 for the numerical values). The estimated error is ±0.05 eV. Data from this paper apart from $[NO_3]^-$,[5] $[NPf_2]^-$,[5] $[CH_3CO_2]^-$,[14] $[SbF_6]^-$,[13] $[I_3]^-$.[12] Large E_B(N_{cation} 1s) = weak electrostatically interacting anion and small E_B(N_{cation} 1s) = strong electrostatically interacting anion.

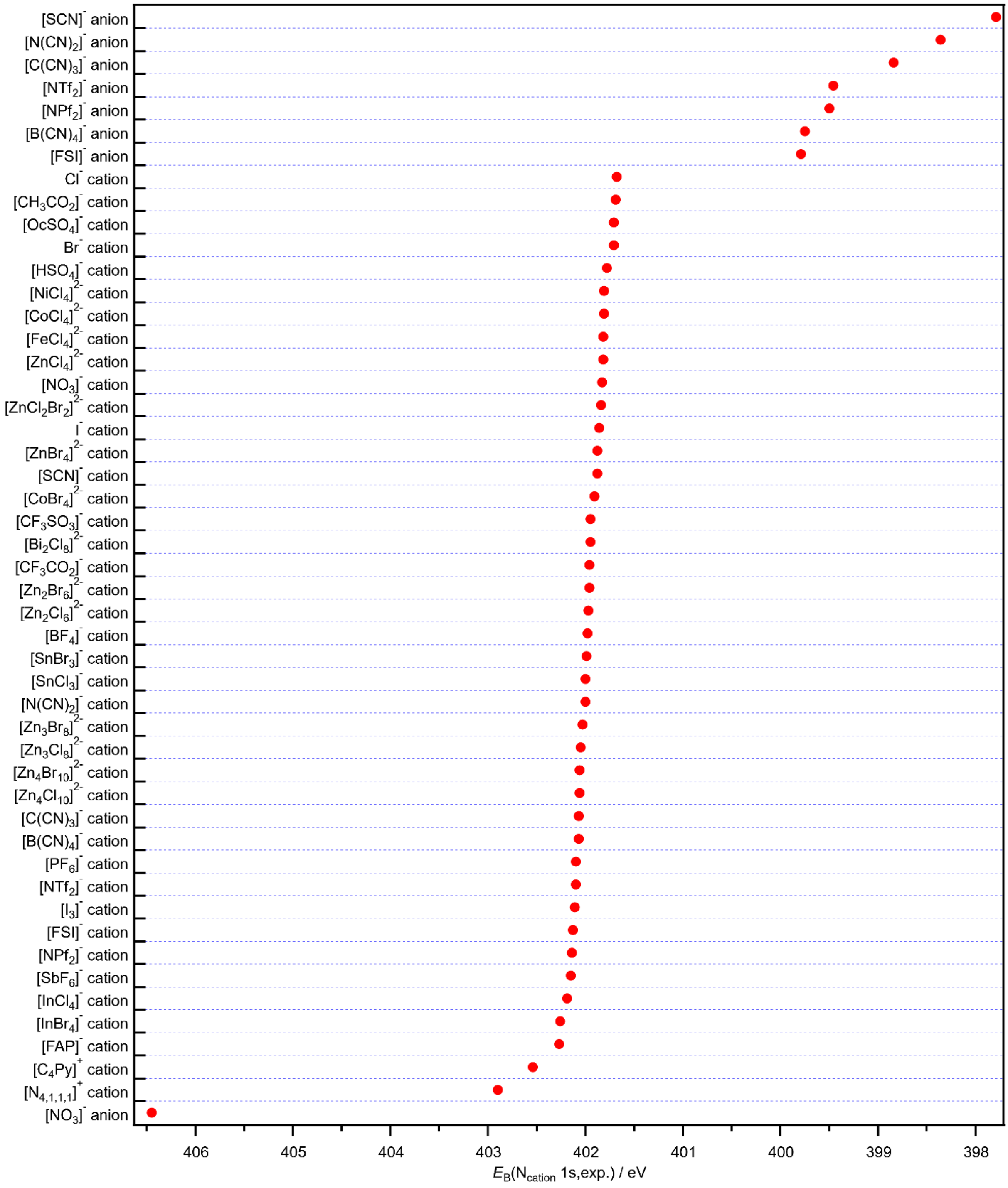

Figure S13. E_B(N 1s) for eight different anions and 41 different cations (see ESI Table S5 and Table S6 for the numerical values). The estimated error is ±0.05 eV. Data from our group (here and references [3, 4]) apart from $[NO_3]^-$,[5] $[NPf_2]^-$,[5] $[CH_3CO_2]^-$,[14] $[SbF_6]^-$,[13] $[I_3]^-$.[12]

5. Results. Experimental versus calculated core XPS

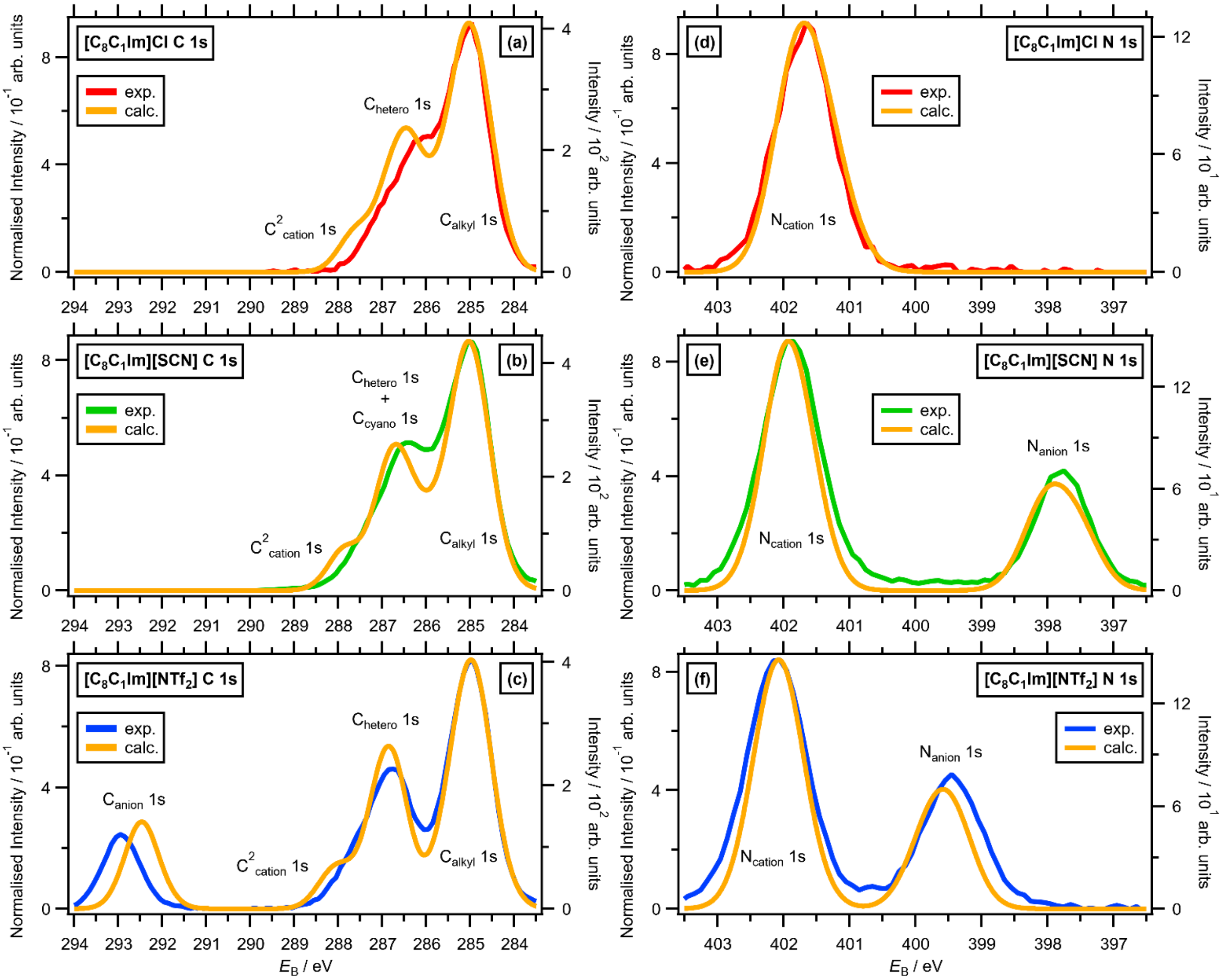

Figure S14. Experimental and calculated (for three configurations of each IL, FWHM = 0.7 eV) C 1s XPS for: (a) $[C_8C_1Im]Cl$, (b) $[C_8C_1Im][SCN]$, (c) $[C_8C_1Im][NTf_2]$. Experimental and calculated (for three configurations of each IL, FWHM = 0.7 eV) N 1s XPS for: (d) $[C_8C_1Im]Cl$, (e) $[C_8C_1Im][SCN]$, (f) $[C_8C_1Im][NTf_2]$.

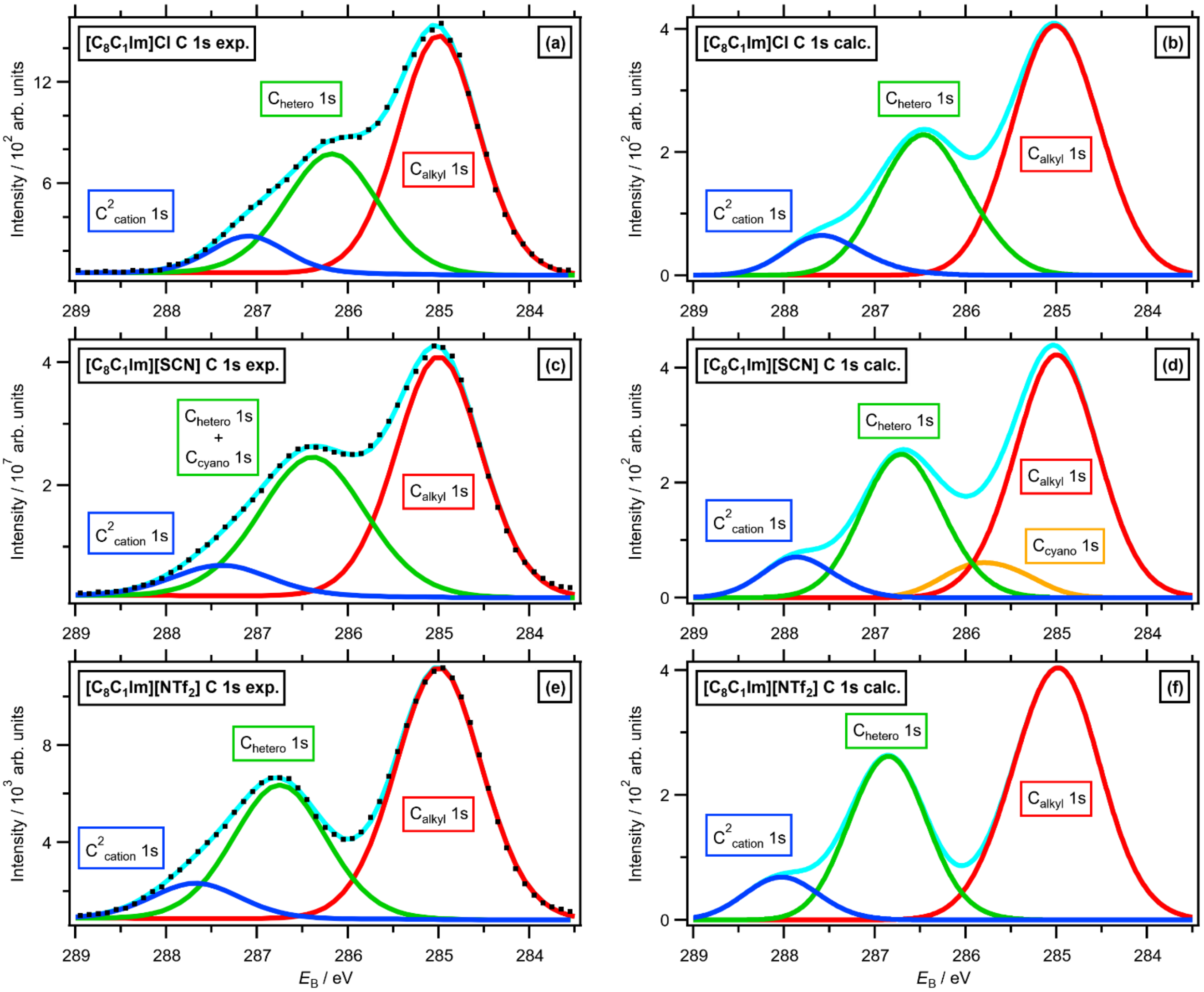

Figure S15. Experimental C 1s XPS for: (a) [C_8C_1Im]Cl, (b) [C_8C_1Im][SCN], (c) [C_8C_1Im][NTf_2]. Calculated (for three configurations of each IL, FWHM = 0.7 eV) C 1s XPS for: (d) [C_8C_1Im]Cl, (e) [C_8C_1Im][SCN], (f) [C_8C_1Im][NTf_2].

6. Results. Linear correlations of E_B and ESP

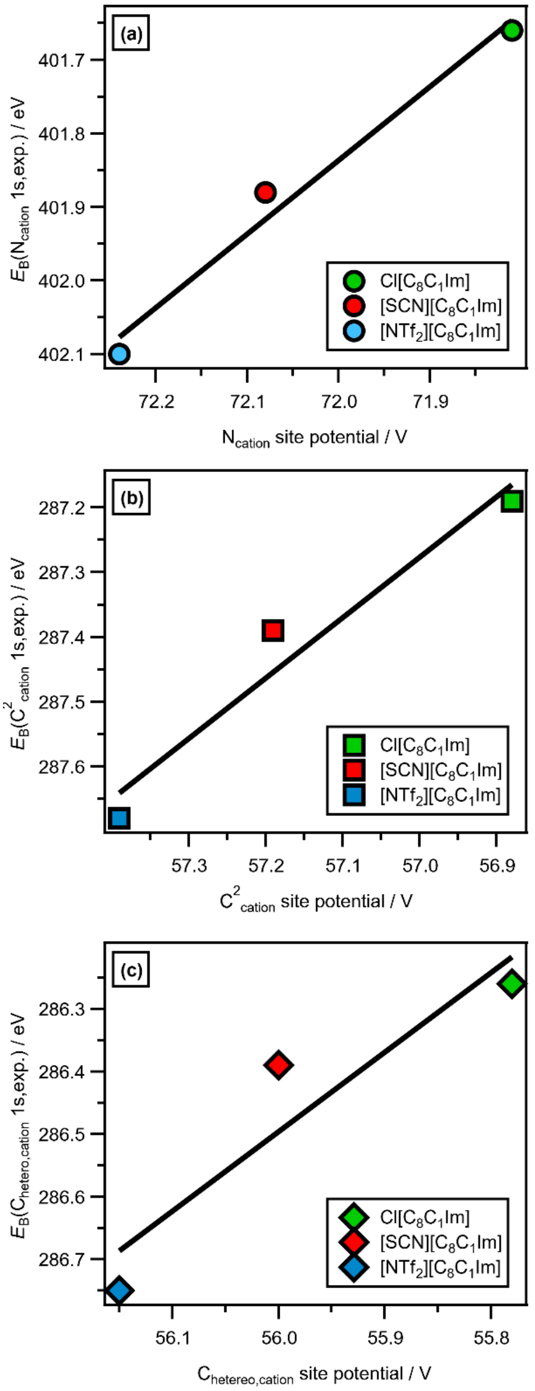

Figure S16. Calculated XPS E_B and site potential data for $[C_8C_1Im][A]$ where $[A]^-$ = Cl^-, $[SCN]^-$ and $[NTf_2]^-$: (a) average $E_B(N_{cation}$ 1s,calc.) (for three configurations of each IL) versus average N_{cation} site potential (for three configurations of each IL); (b) average $E_B(C^2_{cation}$ 1s,calc.) (for three configurations of each IL) versus average C^2_{cation} site potential (for three configurations of each IL); (c) average $E_B(C_{hetero}$ 1s,calc.) (for three configurations of each IL) versus average C_{hetero} site potential (for three configurations of each IL).

7. Results. Proving that size does not matter strongly to anion interaction strength

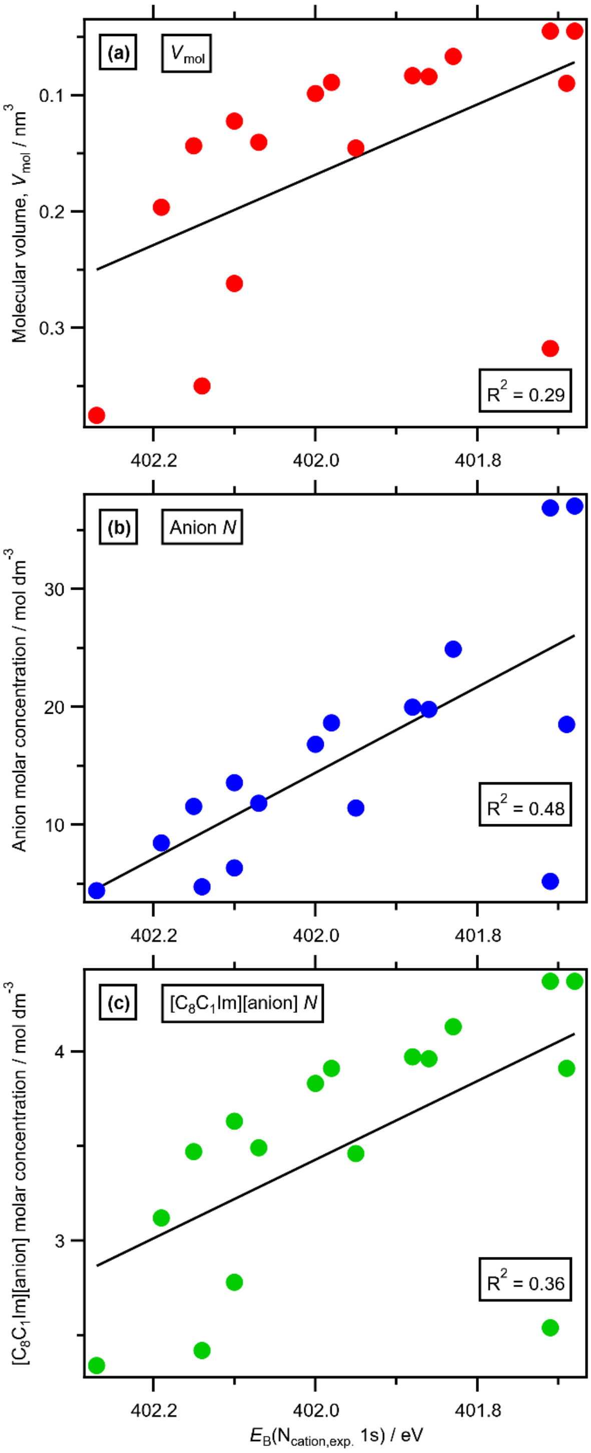

Figure S17. Anion properties plotted against E_B(N_{cation} 1s,exp.): (a) Calculated anion molecular volume, V_{mol}, taken from reference [10] versus E_B(N_{cation} 1s,exp.) for 17 ILs (same plot as Figure 6c in main manuscript); (b) calculated anion molar concentration calculated from data in reference [10] versus E_B(N_{cation} 1s,exp.) for 17 ILs; (c) calculated [C_8C_1Im][anion] molar concentration calculated from data in reference [10] versus E_B(N_{cation} 1s,exp.) for 17 ILs (ESI Table S5 for values used).

8. Results. E_B(O_{anion} 1s) versus E_B(N_{cation} 1s)

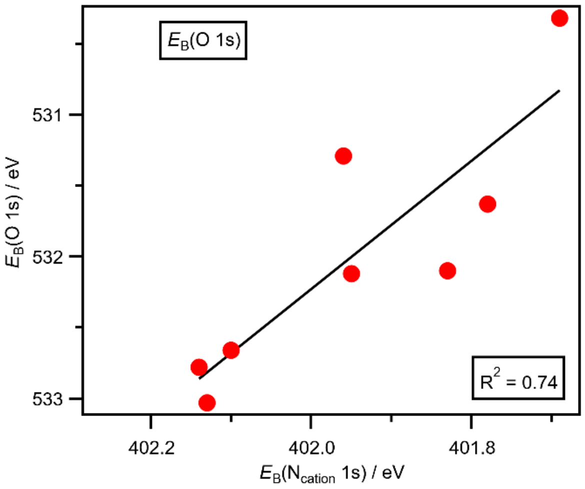

Figure S18. E_B comparisons from experimental XPS for ILs. Experimental E_B(N_{cation} 1s) plotted against other experimental E_B for the same ILs: E_B(O_{anion} 1s) versus E_B(N_{cation} 1s) for eight O-containing ILs (ESI Table S5 for values used).

9. Results. $[A]^-$-dependent $[A]^-$-$[C]^+$ interactions

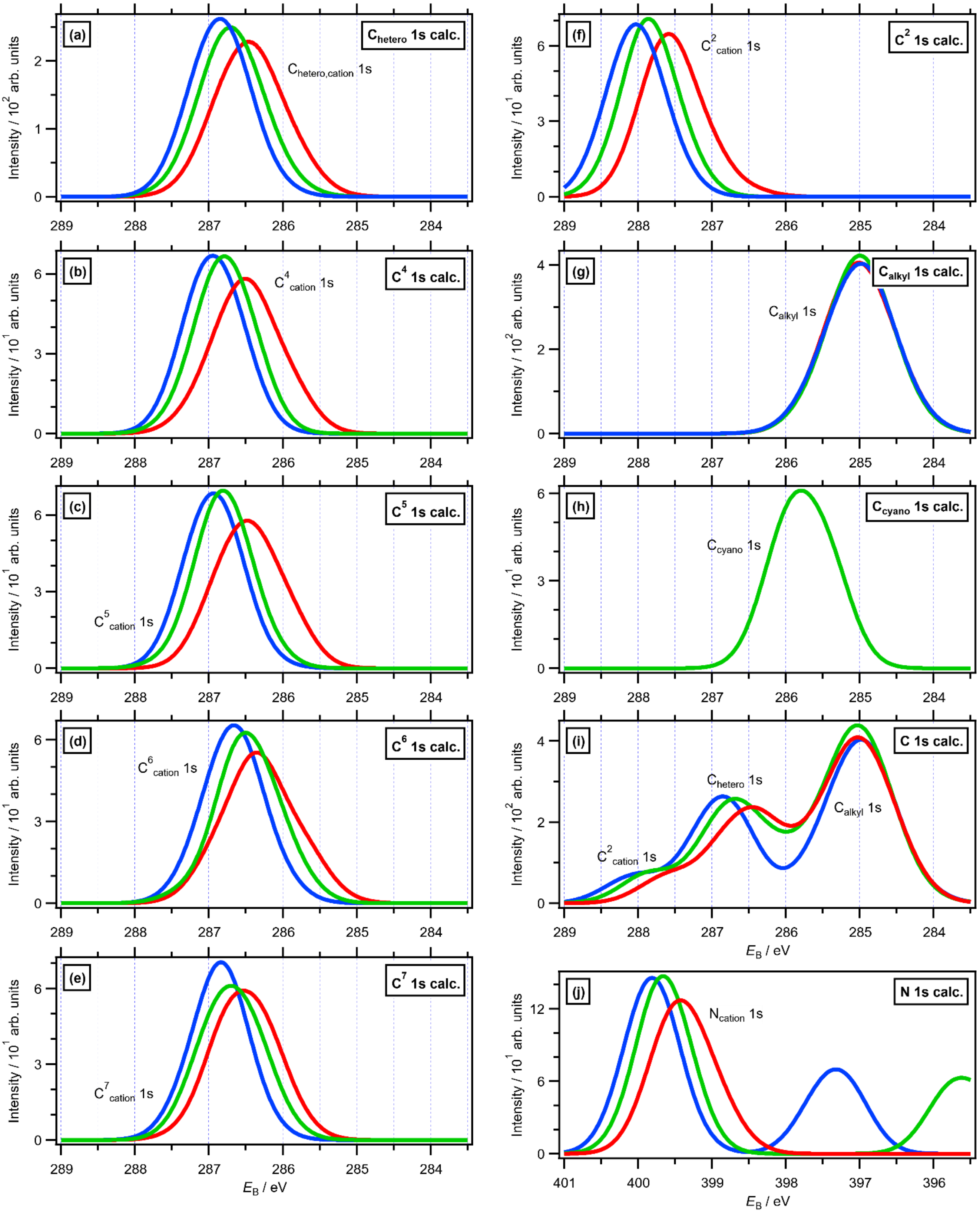

Figure S19. Calculated XPS for $[C_8C_1Im][A]$ where $[A]^-$ = Cl^-, $[SCN]^-$ and $[NTf_2]^-$ for three configurations of each IL (FWHM = 0.7 eV): (a) C_{hetero} 1s XPS, (b) C^4 1s XPS, (c) C^5 1s XPS, (d) C^6 1s XPS, (e) C^7 1s XPS, (f) C^2 1s XPS, (g) C_{alkyl} 1s XPS, (h) C_{cyano} 1s XPS, (i) C_{total} 1s XPS, (j) N_{total} 1s XPS.

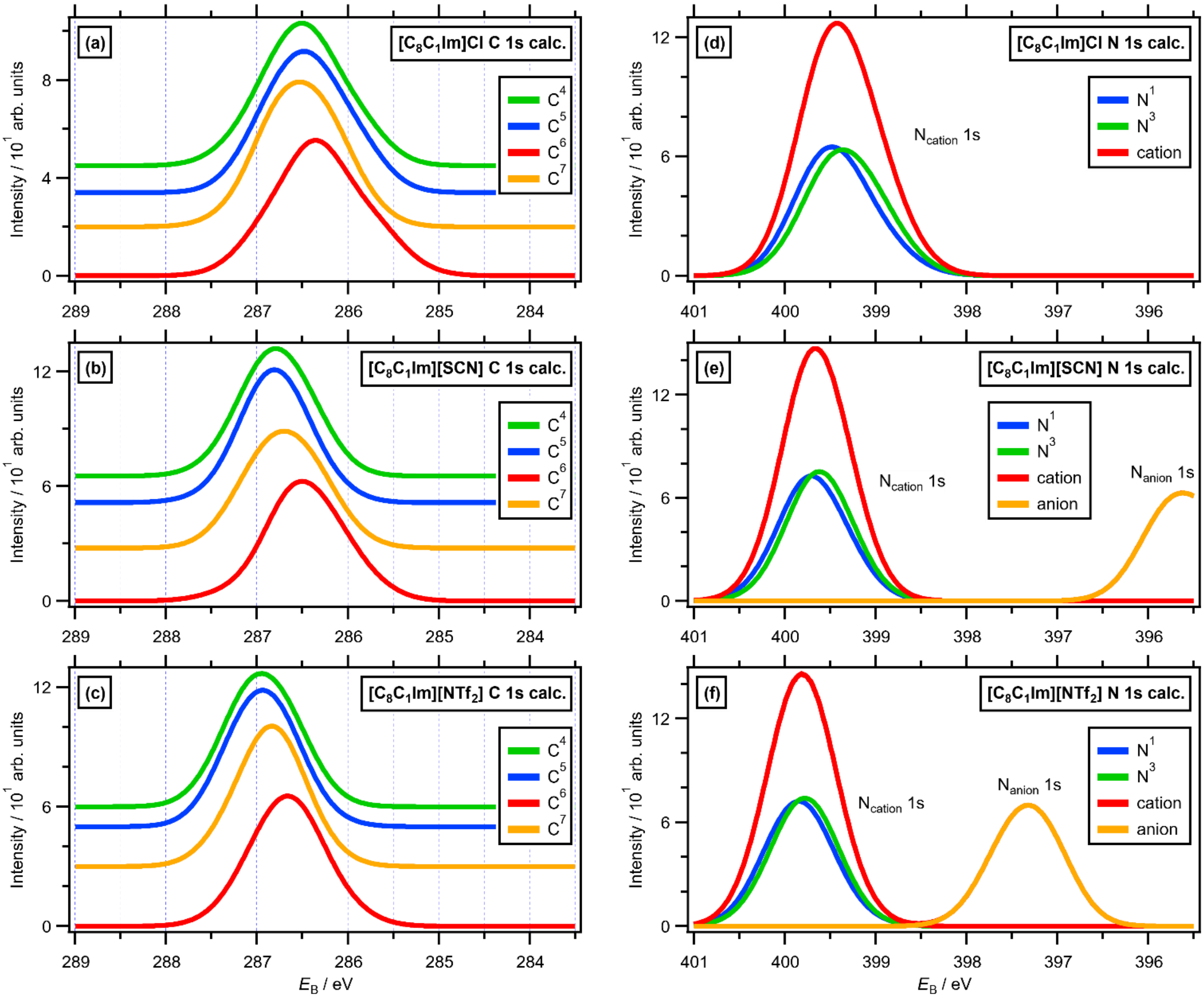

Figure S20. Calculated XPS for $[C_8C_1Im][A]$ where $[A]^-$ = Cl^-, $[SCN]^-$ and $[NTf_2]^-$ for three configurations of each IL (FWHM = 0.7 eV): (a) C_{hetero} 1s XPS breakdown for $[C_8C_1Im]Cl$, (b) C_{hetero} 1s XPS breakdown for $[C_8C_1Im][SCN]$, (c) C_{hetero} 1s XPS breakdown for $[C_8C_1Im][NTf_2]$, (d) N_{cation} 1s XPS breakdown for $[C_8C_1Im]Cl$, (e) N_{cation} 1s XPS breakdown and N_{anion} 1s for $[C_8C_1Im][SCN]$, (f) N_{cation} 1s XPS breakdown and N_{anion} 1s for $[C_8C_1Im][NTf_2]$.

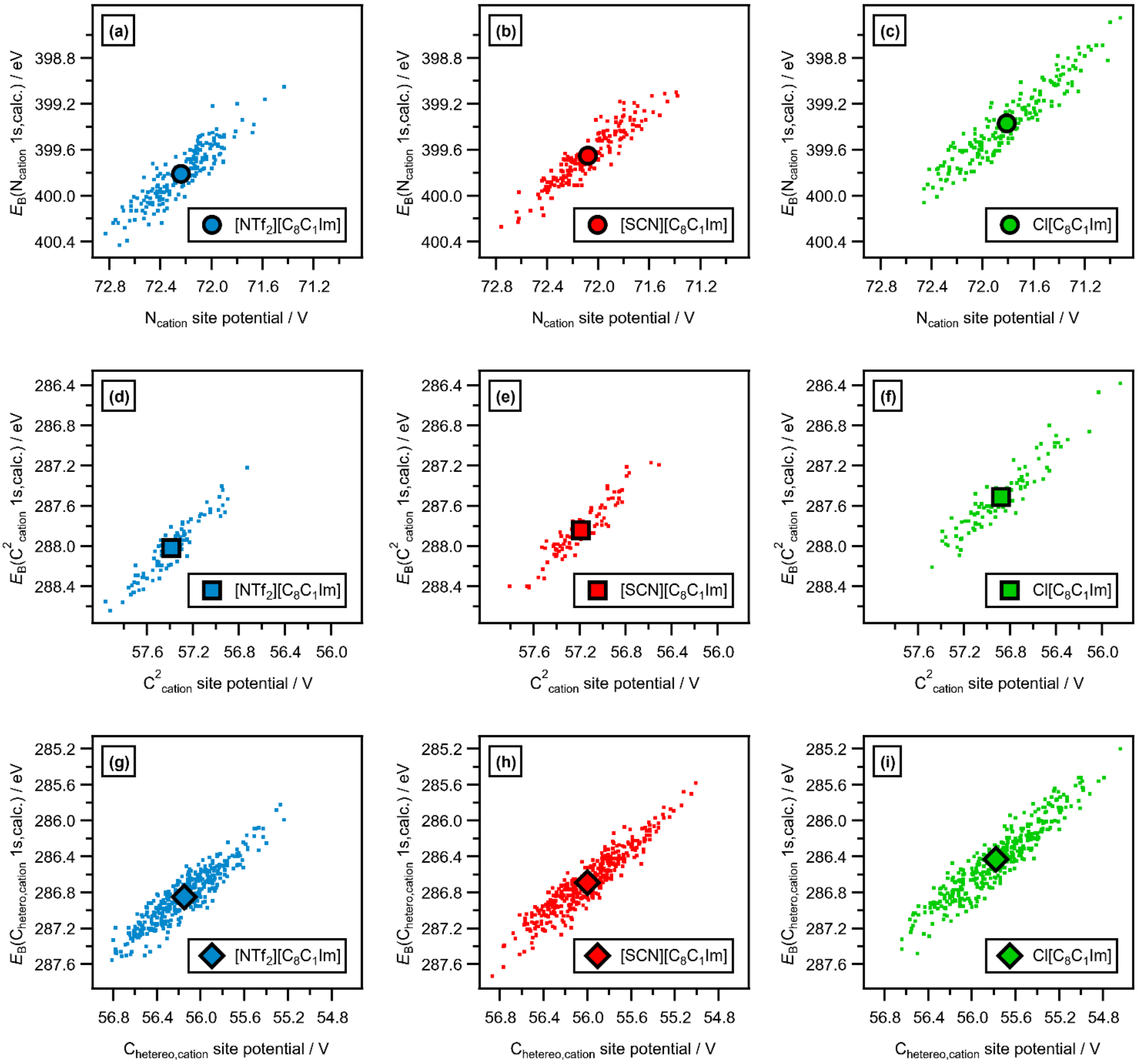

Figure S21. Calculated XPS E_B and site potential data for $[C_8C_1Im][A]$ where $[A]^-$ = Cl^-, $[SCN]^-$ and $[NTf_2]^-$ (both all individual atoms and the average for three configurations of each IL). E_B(N_{cation} 1s,calc.) versus N_{cation} site potential for: (a) $[C_8C_1Im][NTf_2]$; (b) $[C_8C_1Im][SCN]$; (c) $[C_8C_1Im]Cl$. E_B(C^2 1s,calc.) versus C^2 site potential for: (d) $[C_8C_1Im][NTf_2]$; (e) $[C_8C_1Im][SCN]$; (f) $[C_8C_1Im]Cl$. E_B(C_{hetero} 1s,calc.) versus C_{hetero} site potential for: (g) $[C_8C_1Im][NTf_2]$; (h) $[C_8C_1Im][SCN]$; (i) $[C_8C_1Im]Cl$.

10. Results. Anion-cation and anion-neutral molecule interactions

Table S7. Data used for correlations in Figure 7

		DN (ref. [15, 16])	$-E_{HB}$ (ref. [17])	DN (ref. [18])	DN (ref. [15])	β (ref. [19])	β (ref. [20])	β (ref. [21])	β (ref. [22])
**IL**	**$E_B(N_{cation}$ 1s) / eV**	**Figure 7a**	**Figure 7b**	**Figure 7c**	**Figure 7d**	**Figure 7e**	**Figure 7f**	**Figure 7g**	**Figure 7h**
$[C_8C_1Im]Cl$	401.68	10.31	30.72	69.2		0.98	0.94	0.77	12.1
$[C_8C_1Im][CH_3CO_2]$	401.69	10.27	40.17	43.3	11.32	0.85	1.05	1.30	15.0
$[C_4C_1Im][OcSO_4]$	401.71								
$[C_8C_1Im]Br$	401.71	9.94	25.60			0.89	0.74		10.6
$[C_8C_1Im][HSO_4]$	401.78								10.4
$[C_8C_1Im]_2[NiCl_4]$	401.81								
$[C_8C_1Im]_2[CoCl_4]$	401.81								
$[C_8C_1Im]_2[FeCl_4]$	401.82								
$[C_8C_1Im]_2[ZnCl_4]$	401.82								
$[C_8C_1Im][NO_3]$	401.83	9.57	24.21		7.58	0.80		0.67	10.7
$[C_8C_1Im]_2[ZnCl_2Br_2]$	401.84								
$[C_6C_1Im]I$	401.86	9.68	19.97	59.3	7.59	0.81		0.44	8.9
$[C_8C_1Im]_2[ZnBr_4]$	401.88								
$[C_8C_1Im][SCN]$	401.88	9.15	17.01	45.9	7.86	0.71		0.64	
$[C_8C_1Im]_2[CoBr_4]$	401.91								
$[C_8C_1Im][TfO]$	401.95	8.91	17.11	20.4	1.57	0.64	0.49		9.4
$[C_8C_1Im]_2[Bi_2Cl_8]$	401.95								
$[C_8C_1Im][CF_3CO_2]$	401.96	9.74	24.38		6.23	0.74			
$[C_8C_1Im]_2[Zn_2Br_6]$	401.96								
$[C_8C_1Im]_2[Zn_2Cl_6]$	401.97								
$[C_8C_1Im][BF_4]$	401.98	8.64	9.79	7.3	-2.38	0.63	0.41	0.36	
$[C_8C_1Im][SnBr_3]$	401.99								
$[C_8C_1Im][SnCl_3]$	402.00	9.24		39.4					
$[C_4C_1Im][N(CN)_2]$	402.00	8.96	22.6	37.8	5.66	0.71	0.60		
$[C_8C_1Im]_2[Zn_3Br_8]$	402.03								
$[C_8C_1Im]_2[Zn_3Cl_8]$	402.05								
$[C_8C_1Im]_2[Zn_4Br_{10}]$	402.06								
$[C_8C_1Im]_2[Zn_4Cl_{10}]$	402.06								
$[C_8C_1Im][C(CN)_3]$	402.07		16.73	26.1		0.54			
$[C_6C_1Im][B(CN)_4]$	402.07		12.48	20.3		0.46			
$[C_8C_1Im][NTf_2]$	402.10	8.56	9.86	11.2	-3.44	0.47	0.29		7.3
$[C_4C1Im][PF_6]$	402.10	8.38	2.88	-6.2	-4.21	0.53	0.21	0.27	7.0

$[C_8C_1Im][I_3]$	402.11	8.66	2.99						
$[C_8C_1Im][FSI]$	402.13								
$[C_8C_1Im][NPf_2]$	402.14								
$[C_8C_1Im][SbF_6]$	402.15		1.65	4.7		0.42	0.15		
$[C_8C_1Im][InCl_4]$	402.19								
$[C_8C_1Im][InBr_4]$	402.26								
$[C_2C_1Im][FAP]$	402.27		0.74	-12.3		0.31			

Table S8. Linear fitting data from Figure 7. Coefficient values ± one standard deviation for *y* = *a* + *bx*

**Type of data**	**Figure**	***y* = *a* + *bx* data**
electron donor number measured from the chemical shifts δ(H) of the C^2-H proton by ^1H NMR spectroscopy of $[C_4C_1Im][A]$ in the molecular solvent CD_2Cl_2 [15, 16]	7a	a = 1584.8 ± 204<br>b = -3.9199 ± 0.508
hydrogen-bond basicity calculated using ion pairs in COSMO-RS (COnductor-like Screening MOdel for Real Solvents)[17]	7b	a = 23549 ± 2.89e+03<br>b = -58.543 ± 7.19
anion electron donor numbers measured from the chemical shift $\delta(Na^+)$ by ^{23}Na NMR spectroscopy of $Na[ClO_4]$ dissolved in $[C_4C_1Im][A]$ neat ionic liquids[18]	7c	a = 49666 ± 8.94e+03<br>b = -123.49 ± 22.2
anion electron donor numbers measured from the peak shift of the copper complex [Cu(acetylacetonate)(tetramethylethylenediamine)]$[ClO_4]$ by UV-vis spectroscopy in $[C_4C_1Im][A]$ neat ionic liquids[15]	7d	a = 15350 ± 3.05e+03<br>b = -38.181 ± 7.58
Kamlet-Taft hydrogen-bond acceptor numbers using UV-vis spectroscopy comparisons of two different neutral dye molecules in $[C_4C_1Im][A]$ neat ionic liquids[19]	7e	a = 424.12 ± 32.2<br>b = -1.0535 ± 0.0801
Kamlet-Taft hydrogen-bond acceptor numbers using UV-vis spectroscopy comparisons of two different neutral dye molecules in $[C_4C_1Im][A]$ neat ionic liquids[20]	7f	a = 646.61 ± 79.1<br>b = -1.6074 ± 0.197
hydrogen-bond acceptor numbers measured from the chemical shift δ(F) by ^{19}F NMR spectroscopy of a neutral fluorinated dye molecule dissolved in $[C_4C_1Im][A]$ neat ionic liquids[21]	7g	a = 773.6 ± 225<br>b = -1.9235 ± 0.559
hydrogen-bond acceptor values for anions measured by titration using UV-vis spectroscopy of three different neutral dye molecules in two different organic solvents (MeCN and $CHCl_3$)[22]	7h	a = 5237.8 ± 1.13e+03<br>b = -13.009 ± 2.81

11. References

1. J. X. Mao and K. Damodaran, *Ionics*, 2015, **21**, 1605-1613.
2. J. M. Seymour, E. Gousseva, F. K. Towers Tompkins, L. G. Parker, N. O. Alblewi, C. J. Clarke, S. Hayama, R. G. Palgrave, R. A. Bennett, R. P. Matthews and K. R. J. Lovelock, *Faraday Discuss.*, 2024, DOI: 10.1039/D4FD00029C.
3. R. M. Fogarty, R. G. Palgrave, R. A. Bourne, K. Handrup, I. J. Villar-Garcia, D. J. Payne, P. A. Hunt and K. R. J. Lovelock, *Phys. Chem. Chem. Phys.*, 2019, **21**, 18893-18910.
4. J. M. Seymour, E. Gousseva, A. I. Large, C. J. Clarke, P. Licence, R. M. Fogarty, D. A. Duncan, P. Ferrer, F. Venturini, R. A. Bennett, R. G. Palgrave and K. R. J. Lovelock, *Phys. Chem. Chem. Phys.*, 2021, **23**, 20957-20973.
5. T. Cremer, C. Kolbeck, K. R. J. Lovelock, N. Paape, R. Wölfel, P. S. Schulz, P. Wasserscheid, H. Weber, J. Thar, B. Kirchner, F. Maier and H. P. Steinrück, *Chem.-Eur. J.*, 2010, **16**, 9018-9033.
6. I. J. Villar-Garcia, E. F. Smith, A. W. Taylor, F. L. Qiu, K. R. J. Lovelock, R. G. Jones and P. Licence, *Phys. Chem. Chem. Phys.*, 2011, **13**, 2797-2808.
7. C. Kolbeck, T. Cremer, K. R. J. Lovelock, N. Paape, P. S. Schulz, P. Wasserscheid, F. Maier and H. P. Steinrück, *J. Phys. Chem. B*, 2009, **113**, 8682-8688.
8. C. D. Wagner, L. E. Davis, M. V. Zeller, J. A. Taylor, R. H. Raymond and L. H. Gale, *Surf. Interface Anal.*, 1981, **3**, 211-225.
9. C. Kolbeck, M. Killian, F. Maier, N. Paape, P. Wasserscheid and H. P. Steinrück, *Langmuir*, 2008, **24**, 9500-9507.
10. J. Jacquemin, R. Ge, P. Nancarrow, D. W. Rooney, M. F. Costa Gomes, A. A. H. Pádua and C. Hardacre, *J. Chem. Eng. Data*, 2008, **53**, 716-726.
11. B. B. Hurisso, K. R. J. Lovelock and P. Licence, *Phys. Chem. Chem. Phys.*, 2011, **13**, 17737-17748.
12. S. A. Men, K. R. J. Lovelock and P. Licence, *Chem. Phys. Lett.*, 2017, **679**, 207-211.
13. L. S. Longo, E. F. Smith and P. Licence, *ACS Sustain. Chem. Eng.*, 2016, **4**, 5953-5962.
14. I. J. Villar-Garcia, K. R. J. Lovelock, S. Men and P. Licence, *Chem. Sci.*, 2014, **5**, 2573-2579.
15. M. Holzweber, R. Lungwitz, D. Doerfler, S. Spange, M. Koel, H. Hutter and W. Linert, *Chem.-Eur. J.*, 2013, **19**, 288-293.
16. R. Lungwitz and S. Spange, *New J. Chem.*, 2008, **32**, 392-394.
17. A. F. M. Cláudio, L. Swift, J. P. Hallett, T. Welton, J. A. P. Coutinho and M. G. Freire, *Phys. Chem. Chem. Phys.*, 2014, **16**, 6593-6601.
18. M. Schmeisser, P. Illner, R. Puchta, A. Zahl and R. van Eldik, *Chem.-Eur. J.*, 2012, **18**, 10969-10982.
19. S. Spange, R. Lungwitz and A. Schade, *J. Mol. Liq.*, 2014, **192**, 137-143.
20. M. A. Ab Rani, A. Brandt, L. Crowhurst, A. Dolan, N. H. Hassan, J. P. Hallett, P. A. Hunt, M. Lui, H. Niedermeyer, J. M. Perez-Arlandis, M. Schrems, T. Q. To, T. Welton and R. Wilding, *Phys. Chem. Chem. Phys.*, 2011, **13**, 16831-16840.
21. C. Laurence, S. Mansour, D. Vuluga and J. Legros, *Green Chem.*, 2021, **23**, 1816-1822.
22. S. J. Pike, J. J. Hutchinson and C. A. Hunter, *J. Am. Chem. Soc.*, 2017, **139**, 6700-6706.
